# Supplementary material for: AIMP2-DX2 provides therapeutic interface to control KRAS-driven tumorigenesis
Source: Nat Commun. 2022 May 11;13:2572. doi: 10.1038/s41467-022-30149-2 (PMC9095880; doi:10.1038/s41467-022-30149-2)
Supplement: Supplementary file 1 — Supplementary Information [file 41467_2022_30149_MOESM1_ESM.pdf]

Supplementary information

AIMP2-DX2 provides therapeutic interface to control KRAS-driven tumorigenesis

Dae Gyu Kim, Yongseok Choi, Yuno Lee, Semi Lim, Jiwon Kong, JaeHa Song, Younah Roh, Dipesh S. Harmalkar, Kwanshik Lee, Ja-il Goo, Hye Young Cho, Ameeq Ul Mushtaq, Jihye Lee, Song Hwa Park, Doyeun Kim, Byung Soh Min, Kang Young Lee, Young Ho Jeon, Sunkyung Lee, Kyeong Lee*, & Sunghoon Kim*

*Correspondence
kaylee@dongguk.edu (K.L.), and sunghoonkim@yonsei.ac.kr (S.K.)

Supplementary information include:

Supplementary tables

Supplementary table 1. KRAS mutation status

Supplementary table 2. List of the DX2 top200 interactome

Supplementary table 3. Distance and interaction type of binding residues between DX2 and KRAS4B obtained from the representative snapshot at 533.7 ns

Supplementary figures

Supplementary Fig. 1 DX2-dependent regulation of KRAS, but not of NRAS and HRAS

Supplementary Fig. 2 Role of DX2 in KRAS-mediated cancer progression and positive association of the levels of two proteins in different cancers

Supplementary Fig. 3 Specific binding between DX2 and KRAS, but not NRAS and HRAS

Supplementary Fig. 4 Determination of the domain responsible for the binding of DX2 with KRAS

Supplementary Fig. 5 Determination of the binding mode of DX2 with KRAS

Supplementary Fig. 6 Determination of the KRAS status for DX2 binding

Supplementary Fig. 7 Identification of specific E3 ligase, Smruf2, against KRAS

Supplementary Fig. 8 Smurf2-mediated ubiquitination of KRAS

Supplementary Fig. 9 Analysis of EGF-dependent binding kinetics of KRAS and DX2

Supplementary Fig. 10 Identification of the ubiquitination sites of KRAS

Supplementary Fig. 11 Screening of the DX2-KRAS interaction inhibitor

Supplementary Fig. 12 Suppression of the cell viability in a DX2-dependent manner via BC-DXI-32982

Supplementary Fig. 13 Determination of the suppressive mode of action of BC-DXI-32982 on the DX2-KRAS binding

Supplementary Fig. 14 Scheme 1 for synthesis of BC-DXI-32982 and Biotin-32982 #1[a]

Supplementary Fig. 15 Scheme 2 for synthesis of Biotin-32982 #2[a]

Supplementary Notes

Supplementary Note 1 Pharmacokinetics and in vivo toxicity of BC-DXI-32982

Supplementary Note 2 Procedure of chemical synthesis

Supplementary Table 1. KRAS mutation status

Organ	Cell line	KRAS mutation status
Lung	H69	G12S
	WI-38	WT
	WI-26	WT
	HCC44	G12C
	H1975	WT
	HCC2108	WT
	H1299	WT
	H1666	WT
	H1792	G12C
	Calu-6	Q61K
	H226	WT
	H441	G12V
	A549	G12S
	HCC827	WT
	H1650	WT
	H520	WT
	HCC1588	WT
	H460	Q61H
Colon	HCT-116	G13D
	CCD18CO	WT
	COLO-205	WT
	KM-12	WT
	HCT-8	WT
	SNU-C4	WT
	CaCo2	WT
	LoVo	G13D
	DLD-1	G13D
	SW-403	G12V
	SNU-407	G12D
	H747	G13D
Pancreas	SNU-410	G12V
	BxPC3	WT
	AsPC-1	G12D
	Panc10.05	G12D
	Panc1	G12D
	SU.86.86	G12D
	MIA-PaCa2	G12C
	CFPAC-1	G12V

Supplementary Table 2. List of the DX2 top200 interactome

	Protein	Gene	Relative value of fold change (DX2/EV)
1	Isoform 1 of Heterogeneous nuclear ribonucleoprotein M	HNRNPM	1
2	Tubulin alpha-1A chain	TUBA1A	0.596059113
3	Putative elongation factor 1-alpha-like 3	EEF1A1P5	0.507389163
4	Ubiquitin C splice variant	UBC	0.448275862
5	cDNA FLJ53312, highly similar to Heterogeneous nuclear ribonucleoprotein K	HNRNPK	0.428571429
6	CTP synthase 1	CTPS1	0.428571429
7	Isoform 1 of Polypyrimidine tract-binding protein 1	PTBP1	0.428571429
8	ATP-dependent RNA helicase DDX3X	DDX3X	0.418719212
9	Isoform Somatic-1 of Angiotensin-converting enzyme	ACE	0.379310345
10	cDNA FLJ53366, highly similar to Probable ATP-dependent RNA helicase DDX5	DDX5	0.379310345
11	Actin, cytoplasmic 1	ACTB	0.379310345
12	Isoform 3 of Plasminogen activator inhibitor 1 RNA-binding protein	SERBP1	0.369458128
13	Elongation factor 2	EEF2	0.359605911
14	filamin A, alpha (actin binding protein 280)	FLNA	0.359605911
15	Hornerin	HRNR	0.359605911
16	Vimentin	VIM	0.339901478
17	heterogeneous nuclear ribonucleoprotein H1 (H)	HNRNPH1	0.330049261
18	matrin 3	MATR3	0.330049261
19	Isoform alpha-enolase of Alpha-enolase	ENO1	0.320197044
20	CCT2	CCT2	0.320197044
21	40S ribosomal protein S3	RPS3	0.320197044
22	Isoform 1 of Heat shock cognate 71 kDa protein	HSPA8	0.310344828
23	Tubulin beta chain	TUBB	0.300492611
24	Isoform 1 of Heat shock protein HSP 90-alpha	HSP90AA1	0.300492611
25	Heterogeneous nuclear ribonucleoprotein L	HNRNPL	0.300492611
26	Isoform 2 of Annexin A2	ANXA2	0.290640394
27	NUP153 variant protein (Fragment)	NUP153	0.290640394
28	ATP-citrate synthase	ACLY	0.290640394
29	Peroxiredoxin-1	PRDX1	0.270935961
30	Isoform 1 of RNA-binding protein 14	RBM14	0.270935961
31	GTP-binding nuclear protein Ran	RAN	0.270935961
32	Isoform 1 of Ubiquitin-associated protein 2-like	UBAP2L	0.270935961
33	Isoleucyl-tRNA synthetase, cytoplasmic	IARS1	0.261083744
34	Ubiquitin-like modifier-activating enzyme 1	UBA1	0.261083744
35	Phenylalanyl-tRNA synthetase alpha chain	FARSA	0.251231527
36	Cytochrome b-c1 complex subunit 2, mitochondrial	UQCRC2	0.251231527
37	pyruvate kinase isozymes M1/M2 isoform c	PKM	0.251231527
38	Isoform 1 of Nucleophosmin	NPM1	0.251231527
39	C-1-tetrahydrofolate synthase, cytoplasmic	MTHFD1	0.24137931
40	Isoform Long of Trifunctional purine biosynthetic protein adenosine-3	GART	0.231527094
41	cDNA FLJ54020, highly similar to Heterogeneous nuclear ribonucleoprotein U	HNRNPU	0.221674877
42	eukaryotic initiation factor 4A-I isoform 2	EIF4A1	0.221674877
43	cDNA FLJ52364, highly similar to Heat-shock protein 105 kDa	HSPH1	0.221674877
44	GMP synthase [glutamine-hydrolyzing]	GMPS	0.221674877
45	ATP synthase subunit alpha, mitochondrial	ATP5F1A	0.21182266
46	cDNA FLJ60076, highly similar to ELAV-like protein 1	ELAVL1	0.21182266
47	Guanine nucleotide-binding protein subunit beta-2-like 1	GNB2L1	0.201970443
48	Cip1-interacting zinc finger protein	CIZ1	0.201970443
49	Importin-5	IPO5	0.192118227
50	cDNA FLJ50873, highly similar to DNA replication licensing factor MCM3	MCM3	0.18226601

* The protein is sorted according to the relative value of fold change (DX2/EV)

Supplementary Table 2. List of the DX2 top200 interactome

	Protein	Gene	Relative value of fold change (DX2/EV)
51	Hypoxanthine-guanine phosphoribosyltransferase	HPRT1	0.18226601
52	cDNA FLJ56389, highly similar to Elongation factor 1-gamma	EEF1G	0.18226601
53	Cofilin-1	CFL1	0.18226601
54	Stress-70 protein, mitochondrial	HSPA9	0.172413793
55	Isoform DPI of Desmoplakin	DSP	0.172413793
56	Heat shock protein HSP 90-beta	HSP90AB1	0.162561576
57	Exportin-1	XPO1	0.162561576
58	Peroxiredoxin-2	PRDX2	0.162561576
59	Poly(rC)-binding protein 1	PCBP1	0.15270936
60	ATP synthase subunit beta, mitochondrial	ATP5F1B	0.15270936
61	similar to chaperonin containing TCP1, subunit 8 (theta); chaperonin containing T CP1, subunit 8 (theta)	CCT8	0.15270936
62	Glyceraldehyde-3-phosphate dehydrogenase	GAPDH	0.15270936
63	transgelin 2	TAGLN2	0.15270936
64	Isoform 1 of Cullin-associated NEDD8-dissociated protein 1	CAND1	0.142857143
65	Isoform 1 of Transcription intermediary factor 1-beta	TRIM28	0.142857143
66	Heterogeneous nuclear ribonucleoprotein F	HNRNPF	0.142857143
67	Glycyl-tRNA synthetase	GARS1	0.133004926
68	Tubulin alpha-1C chain	TUBA1C	0.133004926
69	poly(rC)-binding protein 2 isoform b	PCBP2	0.133004926
70	Isoform 1 of Far upstream element-binding protein 2	KHSRP	0.133004926
71	dynamin-like 120 kDa protein, mitochondrial isoform 3	OPA1	0.133004926
72	Importin-7	IPO7	0.133004926
73	Nucleolin	NCL	0.133004926
74	Tubulin beta-2C chain	TUBB2	0.123152709
75	Isoform 4 of Double-stranded RNA-specific adenosine deaminase	ADAR	0.123152709
76	Isoform Cytoplasmic of Glutathione reductase, mitochondrial	GSR	0.123152709
77	T-complex protein 1 subunit delta	CCT4	0.123152709
78	Importin subunit beta-1	KPNB1	0.123152709
79	Isoform Monomeric of Arginyl-tRNA synthetase, cytoplasmic	RARS1	0.113300493
80	elongation factor Tu, mitochondrial precursor	TUFM	0.113300493
81	Glutathione S-transferase Mu 3	GSTM3	0.113300493
82	Cysteine and glycine-rich protein 2	CSRP2	0.113300493
83	Importin subunit alpha-2	KPNA2	0.113300493
84	T-complex protein 1 subunit eta	CCT7	0.113300493
85	Phosphoribosylformylglycinamidine synthase	PFAS	0.113300493
86	Interleukin enhancer-binding factor 2	ILF2	0.113300493
87	Isoform Long of Sodium/potassium-transporting ATPase subunit alpha-1	ATP1A1	0.113300493
88	Isoform 1 of Importin-4	IPO4	0.113300493
89	Isoform 2A of GTPase KRas	KRAS	0.113300493
90	Protein disulfide-isomerase A6	PDIA6	0.103448276
91	Isoform 3 of Exportin-2	CSE1L	0.103448276
92	D-3-phosphoglycerate dehydrogenase	PHGDH	0.103448276
93	Isoform 1 of 14-3-3 protein epsilon	YWHAE	0.103448276
94	chromosome 14 open reading frame 166	c14orf166	0.103448276
95	Adenosylhomocysteinase	AHCY	0.103448276
96	T-complex protein 1 subunit alpha	TCP1	0.103448276
97	Isoform 1 of Adenylate kinase 2, mitochondrial	AK2	0.103448276
98	T-complex protein 1 subunit gamma	CCT3	0.103448276
99	Isoform 1 of Heterogeneous nuclear ribonucleoprotein A3	HNRNPA3	0.103448276
100	Cystatin-A	CSTA	0.103448276

* The protein is sorted according to the relative value of fold change (DX2/EV)

Supplementary Table 2. List of the DX2 top200 interactome

	Protein	Gene	Relative value of fold change (DX2/EV)
101	Fatty acid synthase	FASN	0.103448276
102	Nuclear pore complex protein Nup93	NUP93	0.103448276
103	mRNA export factor	RAE1	0.103448276
104	Isoform 4 of Interleukin enhancer-binding factor 3	ILF3	0.103448276
105	Transitional endoplasmic reticulum ATPase	VCP	0.103448276
106	aldolase A, fructose-bisphosphate	ALDOA	0.093596059
107	ribosomal protein SA pseudogene 9; ribosomal protein SA pseudogene 8; ribosomal protein SA pseudogene 58; ribosomal protein SA pseudogene 19; ribosomal protein SA pseudogene 18; ribosomal protein SA; ribosomal protein SA pseudogene 15; ribosomal protein SA pseudogene 61; ribosomal protein SA pseudogene 29; ribosomal protein SA pseudogene 12	RPSA	0.093596059
108	triosephosphate isomerase isoform 2	TPI1	0.093596059
109	soform 2 of DNA replication licensing factor MCM7	MCM7	0.093596059
110	cDNA FLJ53863, highly similar to Cystathionine beta-synthase	CBS	0.093596059
111	40S ribosomal protein S20	RPS20	0.093596059
112	Isoform 1 of DNA-dependent protein kinase catalytic subunit	PRKDC	0.093596059
113	14-3-3 protein zeta/delta	YWHAZ	0.093596059
114	Isoform 1 of 3'(2'),5'-bisphosphate nucleotidase 1	BPNT1	0.093596059
115	Isoform 2 of Heterogeneous nuclear ribonucleoprotein A1	HNRNPA1	0.093596059
116	Aspartidyl-tRNA synthetaseate, cytoplasmic	DARS1	0.083743842
117	Protease serine 1 (Trypsin-1)	PRSS1	0.083743842
118	DnaJ homolog subfamily C member 7	DNAJC7	0.083743842
119	40S ribosomal protein S4, X isoform	RPS4X	0.083743842
120	Puromycin-sensitive aminopeptidase	NPEPPS	0.083743842
121	Heat shock 70 kDa protein 1A/1B	HSPA1A	0.083743842
122	similar to RAN binding protein 1; RAN binding protein 1	RANBP1	0.083743842
123	protein-L-isoaspartate (D-aspartate) O-methyltransferase	PCMT1	0.083743842
124	Ras-related protein Rab-14	RAB14	0.083743842
125	Glutathione S-transferase P	GSTP1	0.083743842
126	T-complex protein 1 subunit epsilon	CCT5	0.083743842
127	Tubulin beta-3 chain	TUBB3	0.083743842
128	Histone H2A type 1-B/E	H2AC4	0.083743842
129	Isoform 1 of RuvB-like 1	RUVBL1	0.083743842
130	26S proteasome non-ATPase regulatory subunit 2	PSMD2	0.083743842
131	Desmoglein-1	DSG1	0.083743842
132	Putative transferase CAF17, mitochondrial	IBA57	0.083743842
133	Peptidyl-prolyl cis-trans isomerase A	PPIA	0.083743842
134	Pre-mRNA-splicing factor ATP-dependent RNA helicase DHX15	DHX15	0.083743842
135	Parkinson disease (autosomal recessive, early onset) 7	PARK7	0.083743842
136	Leucyl-tRNA synthetase	LARS1	0.073891626
137	14-3-3 protein theta	YWHAQ	0.073891626
138	60S acidic ribosomal protein P0	RPLP0	0.073891626
139	Endoplasmin	HSP90B1	0.073891626
140	40S ribosomal protein S2	RPS2	0.073891626
141	Leucine-rich PPR motif-containing protein, mitochondrial	LRPPRC	0.073891626
142	Serine/threonine-protein phosphatase PP1-beta catalytic subunit	PPP1CB	0.073891626
143	Cell division cycle 2, G1 to S and G2 to M, isoform CRA_a	CDK1	0.073891626
144	Translin	TSN	0.073891626
145	cDNA FLJ32936 fis, clone TESTI2007533, highly similar to RuvB-like 2	RUVBL2	0.073891626
146	Ubiquitinyl hydrolase 1	OTUB1	0.073891626
147	von Hippel-Lindau binding protein 1, isoform CRA_b	VBP1	0.073891626
148	cDNA FLJ55072, highly similar to Succinate dehydrogenase (ubiquinone) flavoprotein subunit, mitochondrial	SDHA	0.073891626
149	glutathione S-transferase mu 2 (muscle)	GSTM2	0.073891626
150	Translation initiation factor eIF-2B subunit alpha	EIF2B1	0.073891626

* The protein is sorted according to the relative value of fold change (DX2/EV)

Supplementary Table 2. List of the DX2 top200 interactome

	Protein	Gene	Relative value of fold change (DX2/EV)
151	Inosine-5'-monophosphate dehydrogenase 2	IMPDH2	0.073891626
152	DnaJ homolog subfamily A member 1	DNAJA1	0.073891626
153	Sideroflexin-1	SFXN1	0.073891626
154	60S ribosomal protein L11	RPL11	0.073891626
155	Dermcidin	DCD	0.073891626
156	peptidase (mitochondrial processing) beta	PMPCB	0.073891626
157	Isoform 1AB of Catenin delta-1	CTNND1	0.073891626
158	Isoform 7 of Titin	TTN	0.064039409
159	Isoform 1 of Mitotic checkpoint protein BUB3	BUB3	0.064039409
160	S-adenosylmethionine synthase isoform type-2	MAT2A	0.064039409
161	High mobility group protein B1	HMGB1	0.064039409
162	Thioredoxin-dependent peroxide reductase, mitochondrial	PRDX3	0.064039409
163	Nuclear migration protein nudC	NUDC	0.064039409
164	ATP synthase gamma chain	ATPC1	0.064039409
165	Isoform B1 of Heterogeneous nuclear ribonucleoproteins A2/B1	HNRNPA2B1	0.064039409
166	Cytochrome b-c1 complex subunit 1, mitochondrial	UQCRC1	0.064039409
167	Multifunctional protein ADE2	PAICS	0.064039409
168	Serine/threonine-protein phosphatase 2A catalytic subunit alpha isoform	PPP2CA	0.064039409
169	Tyrosine-protein kinase CSK	CSK	0.064039409
170	X-ray repair complementing defective repair in Chinese hamster cells 6	XRCC6	0.064039409
171	cDNA FLJ59758, highly similar to S-methyl-5-thioadenosine phosphorylase	MTAP	0.064039409
172	Heterogeneous nuclear ribonucleoprotein H2	HNRNPH2	0.064039409
173	Microtubule-associated protein	MAP4	0.064039409
174	Heterogeneous nuclear ribonucleoprotein A0	HNRNPA0	0.064039409
175	Importin-9	IPO9	0.064039409
176	Proteasome 26S non-ATPase subunit 11 variant (Fragment)	PSMD11	0.064039409
177	Isoform 2 of N-alpha-acetyltransferase 15, NatA auxiliary subunit	NAA15	0.064039409
178	Isoform 1 of Alpha-ketoglutarate-dependent dioxygenase FTO	FTO	0.064039409
179	Voltage-dependent anion-selective channel protein 2	VDAC2	0.064039409
180	Heat shock protein 90Bb	HSP90AB2P	0.064039409
181	Isoform 1 of Zinc finger CCCH-type antiviral protein 1	ZC3HAV1	0.064039409
182	Isoform 2 of Nuclear pore complex protein Nup214	NUP214	0.064039409
183	methionyl-tRNA synthetase	MARS1	0.054187192
184	Bifunctional glutamate/proline--tRNA ligase	EPRS1	0.054187192
185	Isoform 1 of Serine/arginine-rich splicing factor 7	SRSF7	0.054187192
186	Heat shock 70 kDa protein 4	HSPA4	0.054187192
187	cDNA PSEC0175 fis, clone OVARC1000169, highly similar to Protein disulfide-iso merase A3	PDIA3	0.054187192
188	Annexin A5	ANXA5	0.054187192
189	Malate dehydrogenase, mitochondrial	MDH2	0.054187192
190	Stress-induced-phosphoprotein 1	STIP1	0.054187192
191	Nascent polypeptide-associated complex subunit alpha, muscle-specific form	NACA	0.054187192
192	Isoform 2 of TAR DNA-binding protein 43	TARDBP	0.054187192
193	probable ATP-dependent RNA helicase DDX17 isoform 3	DDX17	0.054187192
194	abl-interactor 1	ABI1	0.054187192
195	scaffold attachment factor B1 isoform 2	SAFB	0.054187192
196	Succinate dehydrogenase [ubiquinone] iron-sulfur subunit, mitochondrial	SDHB	0.054187192
197	26S protease regulatory subunit 8	PSMC5	0.054187192
198	regulator of chromosome condensation isoform b	RCC1	0.054187192
199	40S ribosomal protein S9	RPS9	0.054187192
200	F-actin-capping protein subunit alpha-1	CAPZA1	0.054187192

* The protein is sorted according to the relative value of fold change (DX2/EV)

Supplementary Table 3. Distance and interaction type of binding residues between DX2 and KRAS4B obtained from the representative snapshot at 533.7 ns

Receptor Residue	Ligand Residue	Interaction Constituents	Distance	Type
KRAS:LYS184	DX2:THR85	DX2:THR85:HN - KRAS:LYS184:O	1.7667	Conventional
KRAS:LYS184	DX2:THR85	DX2:THR85:HG1 - KRAS:LYS184:O	2.1336	Conventional
KRAS:THR50	DX2:GLN110	DX2:GLN110:HE22 - KRAS:THR50:OG1	2.5371	Conventional
KRAS:LYS5	DX2:HIS84	KRAS:LYS5:HZ2 - DX2:HIS84:NE2	2.0757	Conventional
KRAS:ARG41	DX2:THR117	KRAS:ARG41:HH22 - DX2:THR117:OG1	1.9775	Conventional
KRAS:GLN43	DX2:GLN110	KRAS:GLN43:HE21 - DX2:GLN110:O	2.2793	Conventional
KRAS:GLN70	DX2:SER87	KRAS:GLN70:HE21 - DX2:SER87:O	2.4122	Conventional
KRAS:LYS184	DX2:HIS84	DX2:HIS84:HA - KRAS:LYS184:O	2.8057	Carbon
KRAS:GLU37	DX2:HIS86	DX2:HIS86:HE1 - KRAS:GLU37:OE1	2.4054	Carbon
KRAS:GLN70	DX2:SER87	DX2:SER87:HB2 - KRAS:GLN70:O	2.7711	Carbon
KRAS:ASP38	DX2:LYS129	DX2:LYS129:HE1 - KRAS:ASP38:OD1	2.8895	Carbon
KRAS:ASP38	DX2:ILE132	DX2:ILE132:HA - KRAS:ASP38:OD2	2.9666	Carbon
KRAS:THR50	DX2:GLN110	KRAS:THR50:HB - DX2:GLN110:OE1	2.5785	Carbon
KRAS:LYS184	DX2:VAL83	KRAS:LYS184:HA - DX2:VAL83:O	3.0914	Carbon
KRAS:LYS184	DX2:LEU97	KRAS:LYS184:HE2 - DX2:LEU97:O	2.2692	Carbon
KRAS:CYS185	DX2:HIS84	DX2:HIS84 - KRAS:CYS185	4.5136	Pi-Alkyl
KRAS:TYR40	DX2:ILE132	KRAS:TYR40 - DX2:ILE132	4.961	Pi-Alkyl
KRAS:MET188*	DX2:LYS90	DX2:LYS90:HZ1 - KRAS:MET188:OT2	1.5866	Salt Bridges
KRAS:ASP38	DX2:LYS129	DX2:LYS129:HZ1 - KRAS:ASP38:OD2	1.5702	Salt Bridges
KRAS:LYS182	DX2:GLU94	KRAS:LYS182:HZ2 - DX2:GLU94:OE2	1.6866	Salt Bridges
KRAS:LYS184	DX2:GLU102	KRAS:LYS184:HZ2 - DX2:GLU102:OE2	3.239	Salt Bridges
KRAS:LYS184	DX2:GLU102	KRAS:LYS184:HZ3 - DX2:GLU102:OE1	1.623	Salt Bridges

* KRAS:MET188 is the c-terminal

Supplementary Fig. 1

a

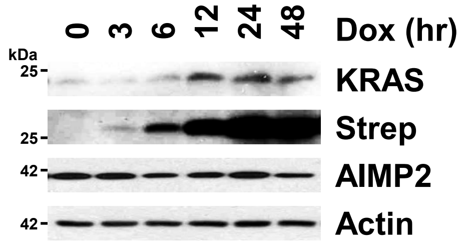

b

RAS isoform	Mutation	Cancer
K4A / 4B	G12C	Lung
	G12D	Pancreas, colon
N	Q61R	Haematopoietic/lymphoid, skin
H	G12V	Bladder

c

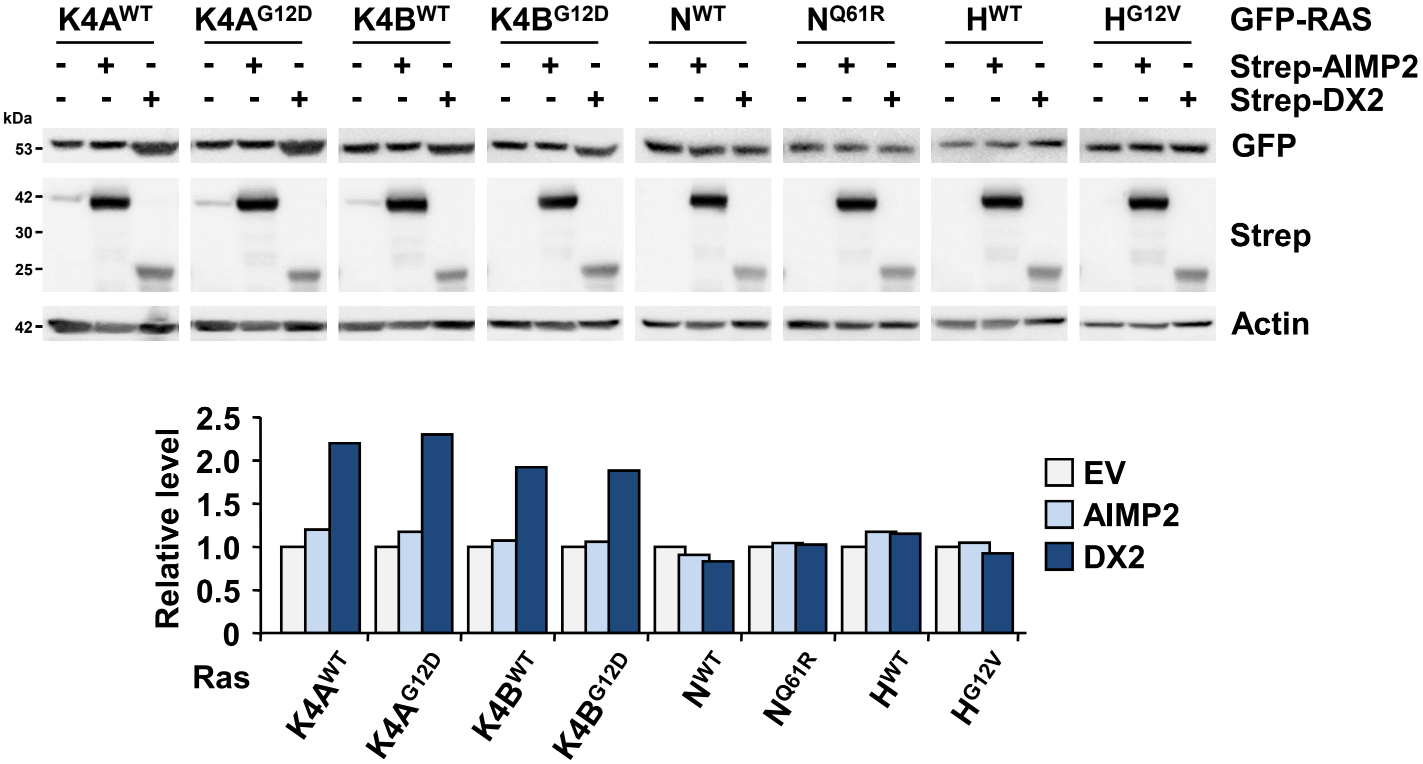

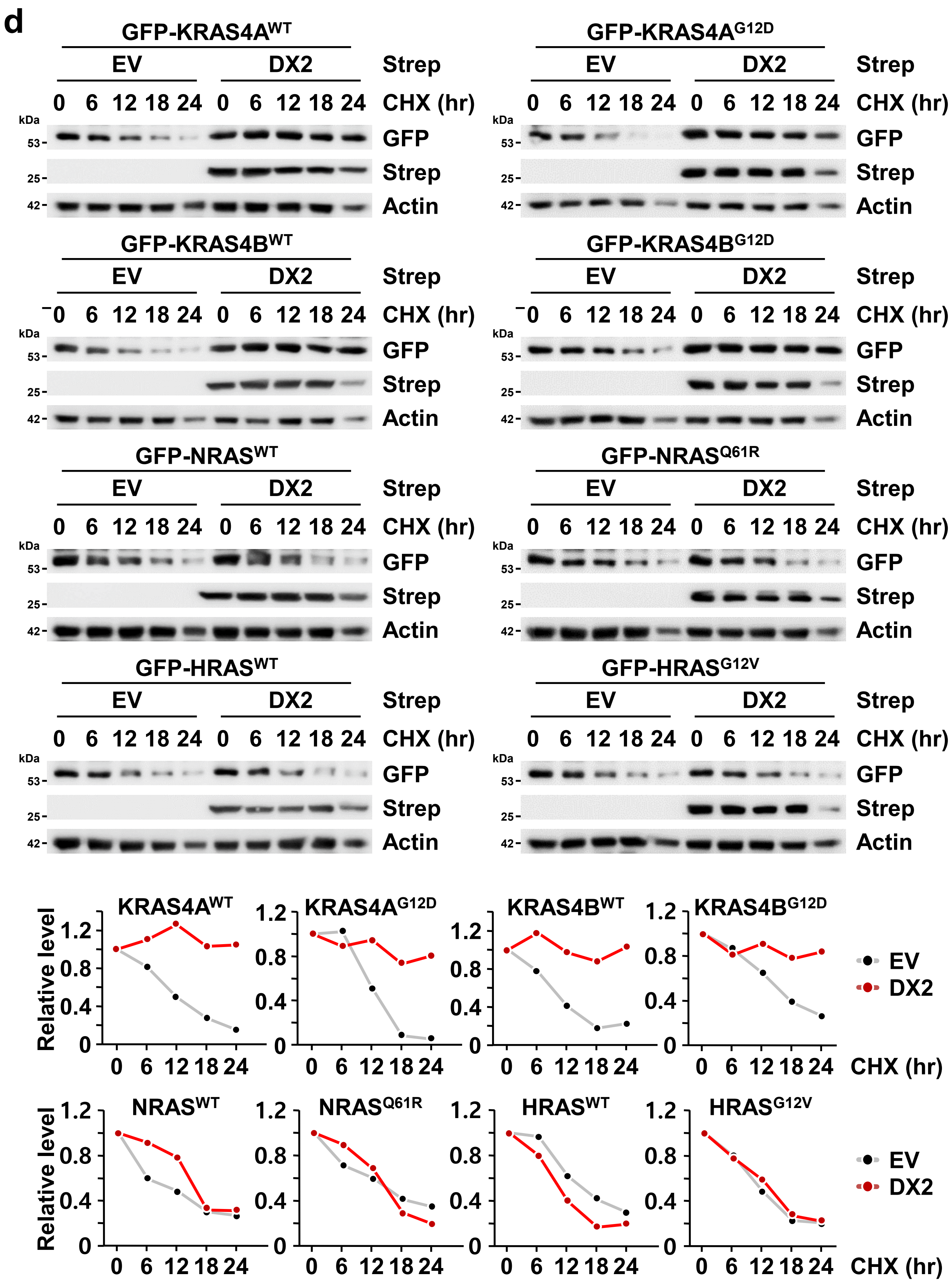
d
GFP-KRAS4A^WT
GFP-KRAS4A^G12D
GFP-KRAS4B^WT
GFP-KRAS4B^G12D
GFP-NRAS^WT
GFP-NRAS^Q61R
GFP-HRAS^WT
GFP-HRAS^G12V
EV
DX2
Strep
0 6 12 18 24 0 6 12 18 24
CHX (hr)
kDa
53-
25-
42-
GFP
Strep
Actin
KRAS4A^WT
KRAS4A^G12D
KRAS4B^WT
KRAS4B^G12D
NRAS^WT
NRAS^Q61R
HRAS^WT
HRAS^G12V
Relative level
0
0.4
0.8
1.2
0 6 12 18 24
CHX (hr)
EV
DX2

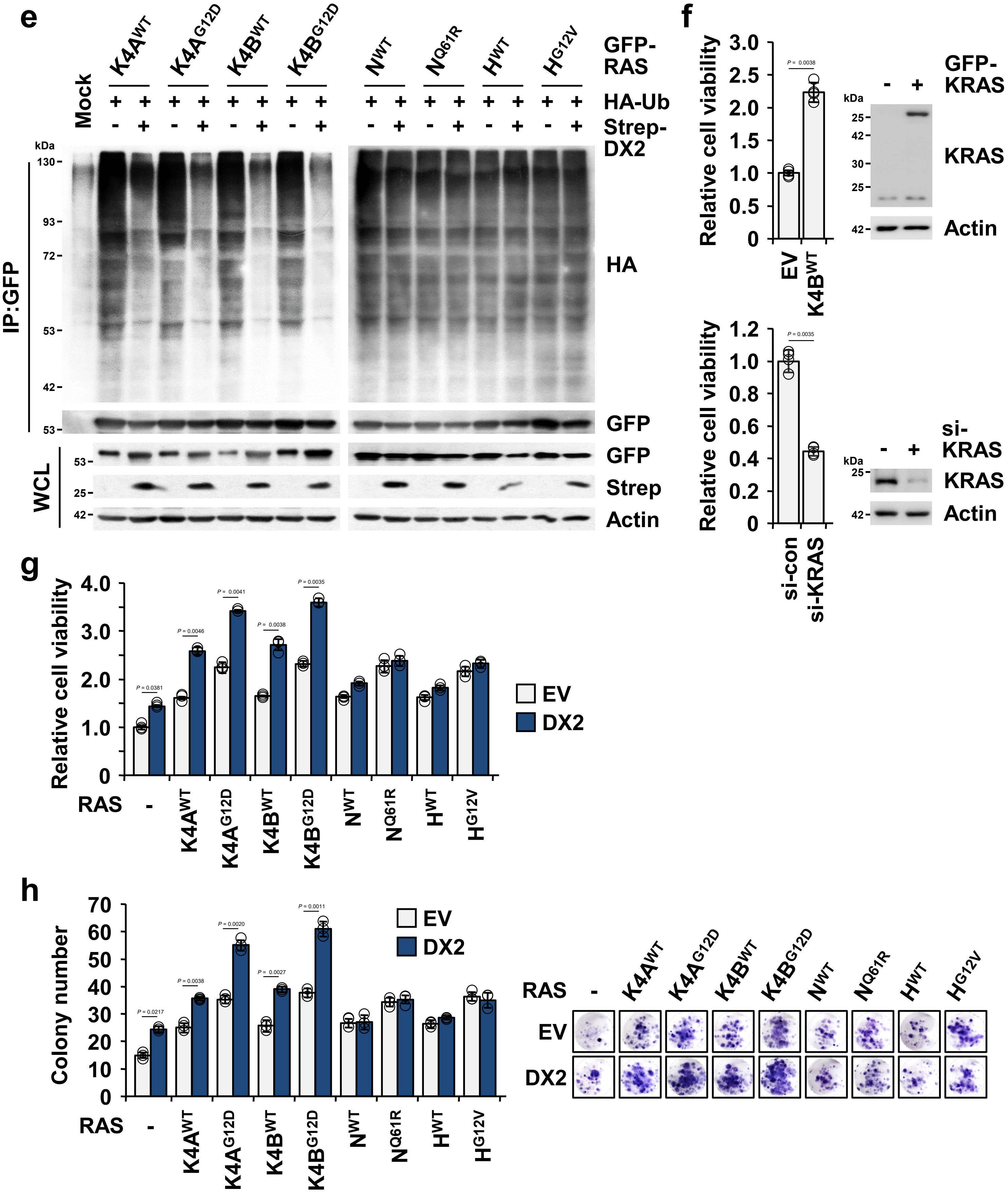
e
Mock
K4A^{WT}
K4A^{G12D}
K4B^{WT}
K4B^{G12D}
N^{WT}
N^{Q61R}
H^{WT}
H^{G12V}
GFP-RAS
HA-Ub
Strep-DX2
kDa
IP:GFP
HA
GFP
WCL
GFP
Strep
Actin
f
Relative cell viability
EV
K4B^{WT}
GFP-KRAS
KRAS
Actin
si-con
si-KRAS
si-KRAS
g
Relative cell viability
RAS
EV
DX2
h
Colony number
RAS
EV
DX2

Supplementary Fig. 1 DX2-dependent regulation of KRAS, but not of NRAS and HRAS. a Strep-DX2-inducible H460 cells were treated by doxycycline (Dox) in a time-dependent manner and cellular levels of DX2, AIMP2, and KRAS were determined by immunoblotting using their specific antibodies. Actin was used as a loading control. **b** RAS isoforms and their different cancer-associated mutations. **c** 293T cells expressing Strep-AIMP2, -DX2, and GFP-RAS isoforms were subjected to SDS-PAGE and immunoblotting. The quantified levels of RAS proteins were presented as a bar graph (bottom). EV denotes “empty vector”. **d** Strep-DX2 and GFP-RAS isoforms in the indicated combination were introduced into A549 cells expressing HSP70. The cells were treated with cycloheximide (CHX) in a time-dependent manner. The levels of RAS proteins were quantified and presented as line graph (bottom). **e** Strep-DX2 was introduced into 293T cells expressing HA-ubiquitin (Ub) and each of the GFP-RAS isoforms. The cells were treated with MG-132 and subjected to immunoprecipitation using the anti-GFP antibody. The amounts of ubiquitinated RAS proteins were determined by immunoblotting with the anti-HA antibody. **f** A549 cells transfected with GFP-KRAS or si-KRAS were subjected to MTT assay to determine the cell viability (left). Exogenous and endogenous levels of KRAS were determined by SDS-PAGE and immunoblotting (right). **g** Strep-DX2 was introduced into CCD18CO cells expressing the indicated GFP-RAS isoforms. Cell viability was measured and represented as above. **h** Cells described above were subjected to an anchorage-independent colony forming assay. The number of colonies is represented as a bar graph (left), and a representative image is shown (right). **c**-**e**, **g**, **h** Results were quantified and are presented as a heap map in Fig. 1d. **f**-**h** All the experiments were independently repeated thrice and error bars denote S.D. Data are presented as mean values +/- S.D. *P*-value is from the two-sided t-test. Source data are provided as a Source data file. **a**, **c**-**e** Results are representative of at least three independent experiments.

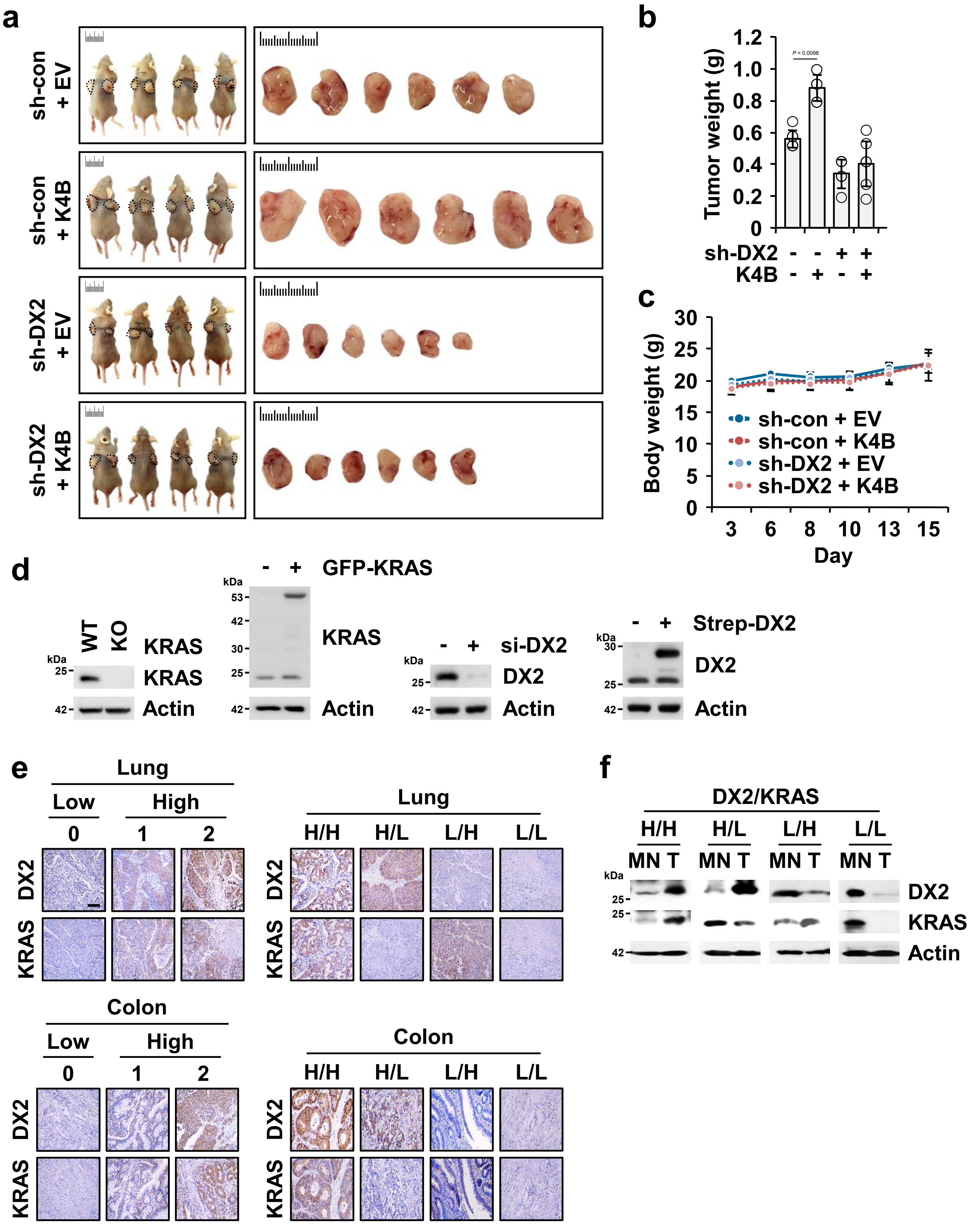
a
sh-con + EV
sh-con + K4B
sh-DX2 + EV
sh-DX2 + K4B
b
Tumor weight (g)
P = 0.0098
sh-DX2
K4B
c
Body weight (g)
sh-con + EV
sh-con + K4B
sh-DX2 + EV
sh-DX2 + K4B
Day
d
WT
KO
KRAS
kDa
Actin
GFP-KRAS
si-DX2
DX2
Strep-DX2
e
Lung
Low
High
H/H
H/L
L/H
L/L
Colon
f
DX2/KRAS
MN T

Supplementary Fig. 2 Role of DX2 in KRAS-mediated cancer progression and positive association of the levels of two proteins in different cancers. a-c H460 cells stably expressing different combinations of KRAS4B and sh-DX2 were xenografted to the backs of nude mice (*n* = 4) and tumor growth was monitored for 15 d (Fig. 1e). Images of the tumor-embedded mice and tumors are shown (**a**). Tumor and body weights were measured and are presented as a bar (**b**) and line (**c**) graph, respectively. All error bars represent the standard deviation (S.D.). *P*-value is from the two-sided t-test. **d** Evaluation of the specific antibody against KRAS and DX2 used for immunohistochemistry (IHC) staining. WT and KO indicate wild type and CRISPR/Cas9-mediated knockout, respectively. Cell lysates were subjected to SDS-PAGE and immunoblotting with the anti-KRAS and DX2 antibodies. **e** Tissue microarray (TMA) slides containing tumors from patients with lung (upper) and colorectal (lower) cancers were subjected to IHC staining to determine the levels of DX2 and KRAS, which were classified as low (0) and high (1 and 2) (left). Representative images for staining are shown (right). The results of the analysis are presented as a Quadrants graph (Fig. 1g). Scale bar = 400 μm. **f** Total protein extracts from the tumor (T) and matched normal (MN) tissues of patients with colorectal cancer were subjected to immunoblotting. The results are shown as a Quadrants graph (Fig. 1h). Source data are provided as a Source data file. **d**, **f** Results are representative of at least three independent experiments.

Supplementary Fig. 3

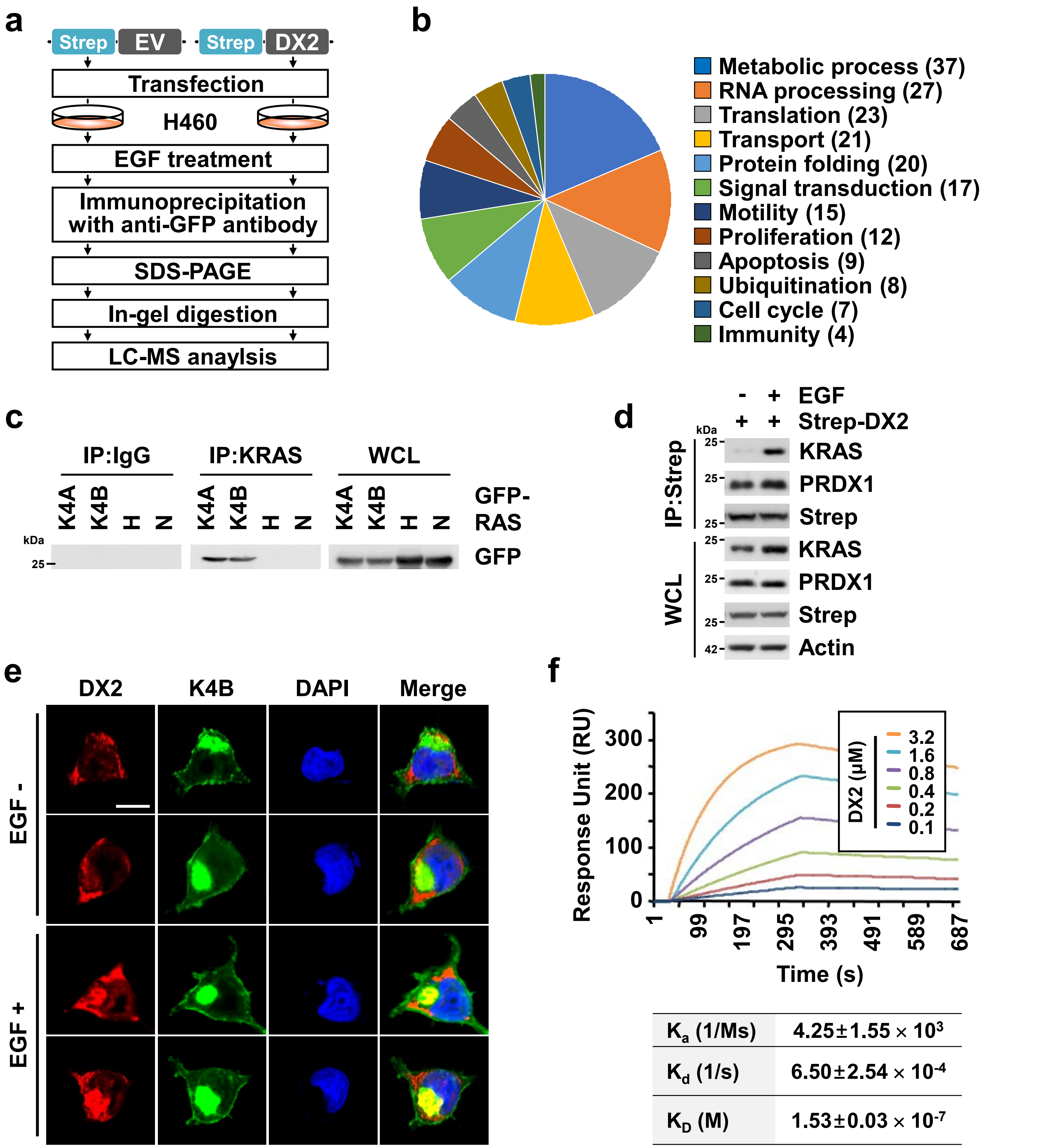

K_a (1/Ms)	$4.25 \pm 1.55 \times 10^3$
K_d (1/s)	$6.50 \pm 2.54 \times 10^{-4}$
K_D (M)	$1.53 \pm 0.03 \times 10^{-7}$

Supplementary Fig. 3 Specific binding between DX2 and KRAS, but not NRAS and HRAS. a Strategy of interactome analysis for identifying DX2-binding proteins upon EGF signaling. **b** Venn diagram showing the ontological classification from DX2-binding top 200 proteins identified by interactome analysis. Number of proteins in each ontology is shown in brackets. **c** Evaluation of specific KRAS antibody available for immunoprecipitation assay. The 293T cells expressing GFP-RAS isoforms were subjected to immunoprecipitation using the specific antibody against KRAS. Immunoprecipitated RAS proteins were determined by SDS-PAGE and immunoblotting using GFP antibody. IgG was used as a negative control for the used antibody. **d** H460 cells expressing Strep-DX2 were treated with EGF for 30 min and subjected to immunoprecipitation. KRAS and PRDX1 co-precipitated with DX2 were determined as above. **e** 293T cells expressing GFP-KRAS4B were treated with EGF, and co-localization (yellow) of endogenous DX2 (red) and KRAS4B (green) was monitored by confocal microscopy. DAPI was used for the staining of nucleus and two representative images are shown. Scale bar = 10 μm. **f** K_D value for the binding affinity between DX2 and KRAS4B as determined by surface plasmon resonance analysis (*n* = 1). Source data are provided as a Source data file. **c**-**d** Results are representative of at least three independent experiments.

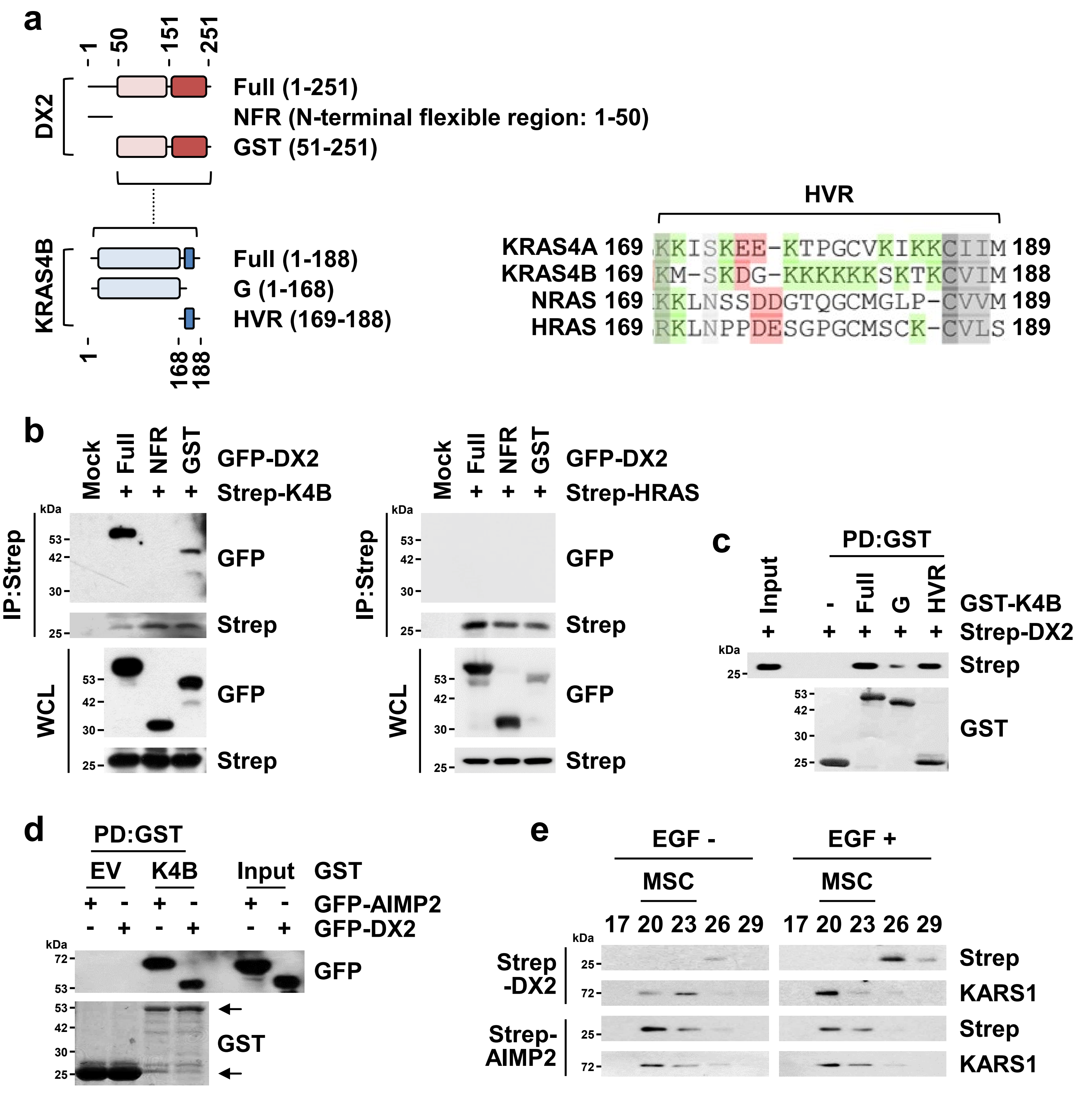
a
DX2
Full (1-251)
NFR (N-terminal flexible region: 1-50)
GST (51-251)
KRAS4B
Full (1-188)
G (1-168)
HVR (169-188)
HVR
KRAS4A 169 KKISKEE-KTPGCVKIKKCIIM 189
KRAS4B 169 KM-SKDG-KKKKKKSKTKCVIM 188
NRAS 169 KKLNSSDDGTQGCMGLP-CVVM 189
HRAS 169 RKLNPPDESGPGCMSCK-CVLS 189
b
Mock Full NFR GST GFP-DX2
+ + + Strep-K4B
+ + + Strep-HRAS
IP:Strep
WCL
GFP
Strep
c
Input
PD:GST
- Full G HVR GST-K4B
+ + + + + Strep-DX2
Strep
GST
d
PD:GST
EV K4B Input GST
GFP-AIMP2
GFP-DX2
GFP
GST
e
EGF -
EGF +
MSC
17 20 23 26 29
Strep-DX2
Strep-AIMP2
Strep
KARS1

Supplementary Fig. 4 Determination of the domain responsible for the binding of DX2 with KRAS. **a** Domains involved in the interaction (marked with brackets) are connected by a dashed line (left). Amino acid sequence alignment of the HVR from four RAS isoforms. Grey, green, and pink colors indicate the sequence similarity, positive and negative charges, respectively (right). **b** 293T cells expressing different GFP-tagged domains of DX2 and Strep-KRAS4B (left) or -HRAS (right) were precipitated using a strep-tactin column. The DX2 domain that co-precipitated with RAS isoforms was detected by immunoblotting with anti-GFP antibody. **c** Different GST-tagged domains of KRAS4B were mixed with cell extracts containing Strep-DX2 and precipitated with glutathione-Sepharose beads. The DX2 domain that co-precipitated with KRAS4B was determined by immunoblotting using anti-strep antibody. GST-KRAS4B domains were confirmed by Coomassie staining. PD means pull-down. **d** Purified GST-KRAS4B mixed with cell lysates containing each of GFP-AIMP2 and -DX2 was precipitated with glutathione-Sepharose beads. Binding of AIMP2 and DX2 to KRAS was determined as above. **e** CCD18CO cells expressing Strep-DX2 or -AIMP2 were treated with EGF for 4 h. Proteins in the cell extracts were separated by gel-filtration chromatography. DX2 and AIMP2 in the indicated fractions were detected by immunoblotting. KARS1 (lysyl-tRNA synthetase1) was monitored as a representative component of the multi-tRNA synthetase complex (MSC). Source data are provided as a Source data file. **b**-**e** Results are representative of at least three independent experiments.

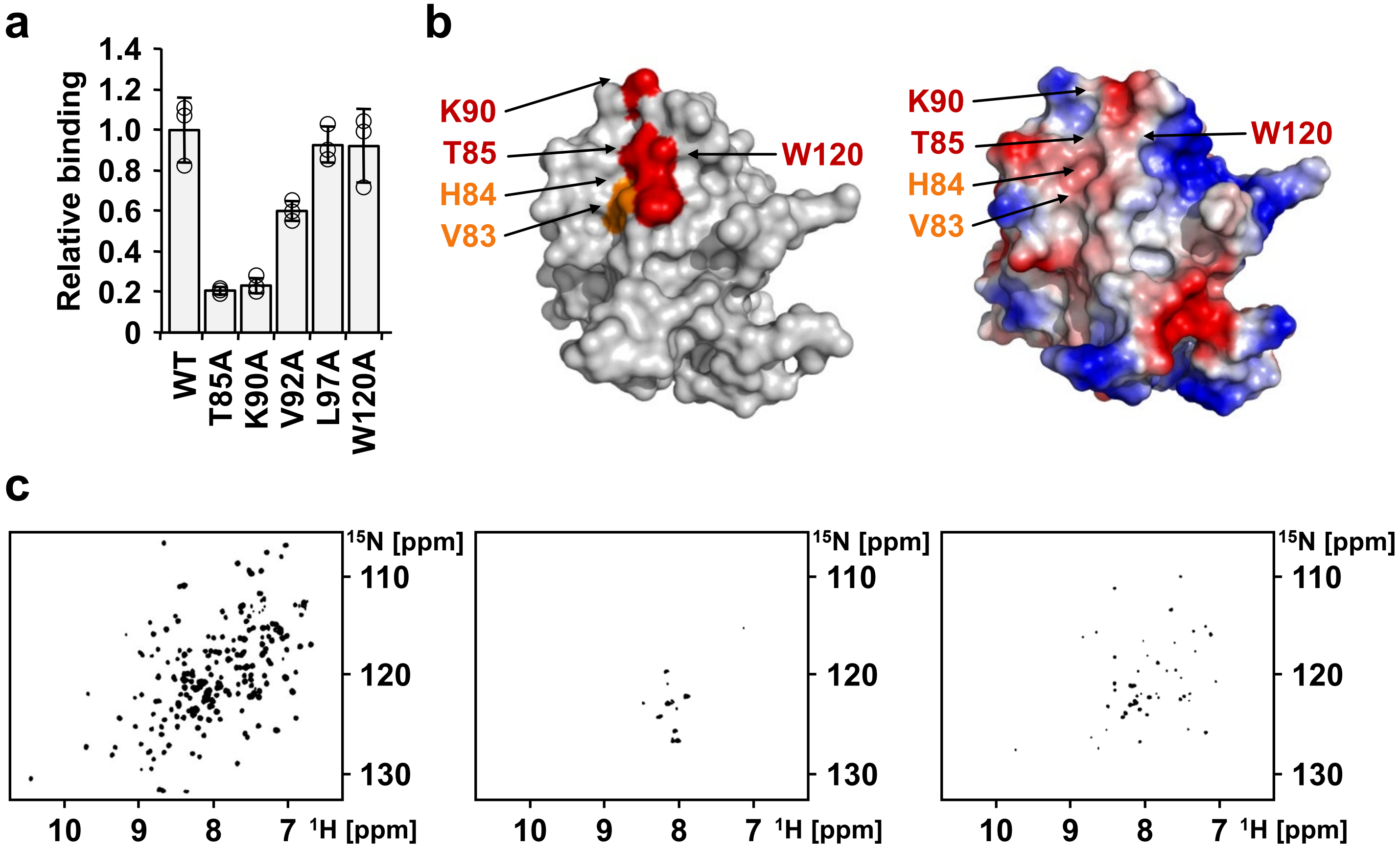
a
Relative binding
1.4
1.2
1.0
0.8
0.6
0.4
0.2
0
WT
T85A
K90A
V92A
L97A
W120A
b
K90
T85
H84
V83
W120
K90
T85
H84
V83
W120
c
^{15}N [ppm]
110
120
130
10
9
8
7
^{1}H [ppm]

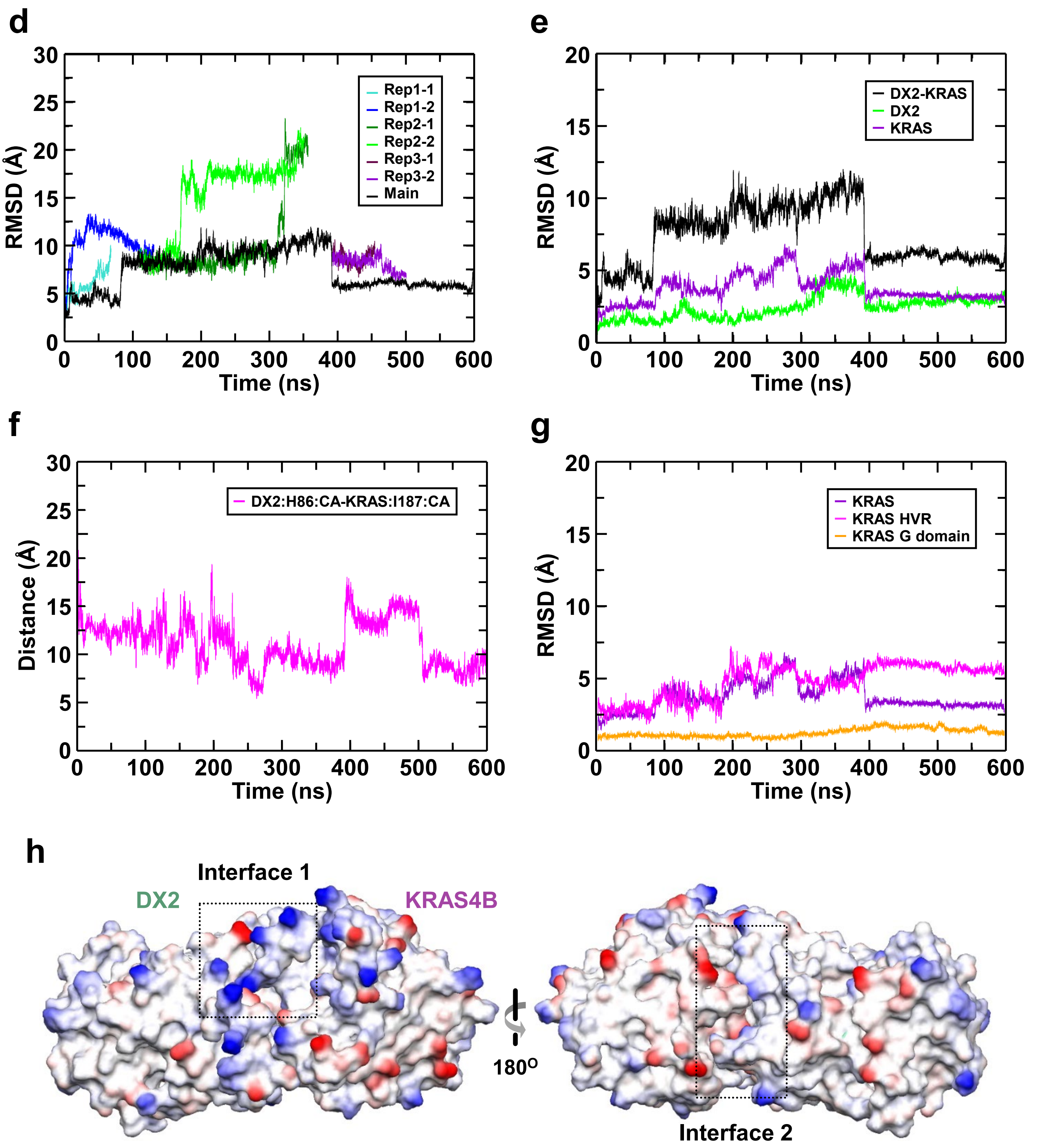
d
RMSD (Å)
Time (ns)
Rep1-1
Rep1-2
Rep2-1
Rep2-2
Rep3-1
Rep3-2
Main
e
RMSD (Å)
Time (ns)
DX2-KRAS
DX2
KRAS
f
Distance (Å)
Time (ns)
DX2:H86:CA-KRAS:I187:CA
g
RMSD (Å)
Time (ns)
KRAS
KRAS HVR
KRAS G domain
h
Interface 1
DX2
KRAS4B
180°
Interface 2

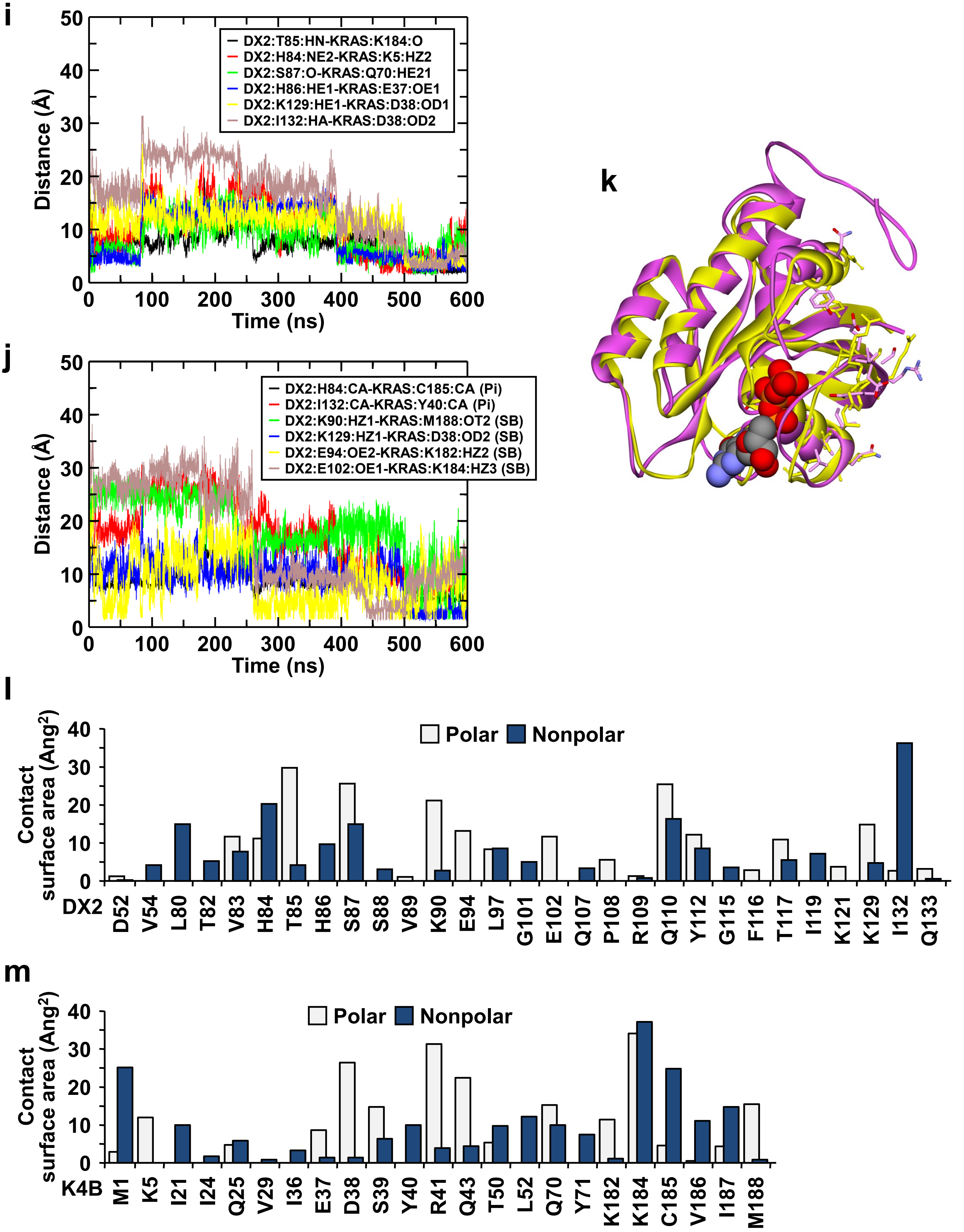
i
Distance (Å)
Time (ns)
DX2:T85:HN-KRAS:K184:O
DX2:H84:NE2-KRAS:K5:HZ2
DX2:S87:O-KRAS:Q70:HE21
DX2:H86:HE1-KRAS:E37:OE1
DX2:K129:HE1-KRAS:D38:OD1
DX2:I132:HA-KRAS:D38:OD2
j
Distance (Å)
Time (ns)
DX2:H84:CA-KRAS:C185:CA (Pi)
DX2:I132:CA-KRAS:Y40:CA (Pi)
DX2:K90:HZ1-KRAS:M188:OT2 (SB)
DX2:K129:HZ1-KRAS:D38:OD2 (SB)
DX2:E94:OE2-KRAS:K182:HZ2 (SB)
DX2:E102:OE1-KRAS:K184:HZ3 (SB)
k
l
Contact surface area (Ang^2)
Polar
Nonpolar
DX2
D52 V54 L80 T82 V83 H84 T85 H86 S87 S88 V89 K90 E94 L97 G101 E102 Q107 P108 R109 Q110 Y112 G115 F116 T117 I119 K121 K129 I132 Q133
m
Contact surface area (Ang^2)
Polar
Nonpolar
K4B
M1 K5 I21 I24 Q25 V29 I36 E37 D38 S39 Y40 R41 Q43 T50 L52 Q70 Y71 K182 K184 C185 V186 I187 M188

n

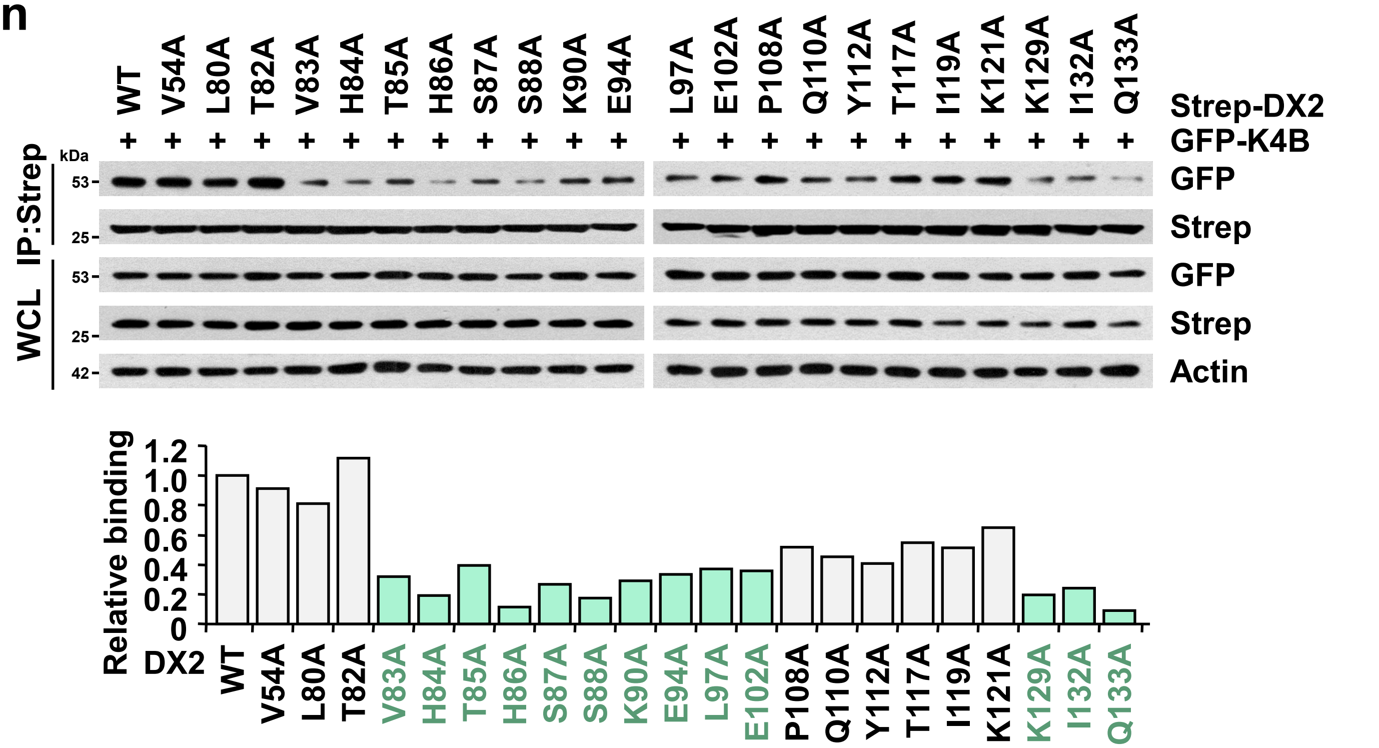
WT
V54A
L80A
T82A
V83A
H84A
T85A
H86A
S87A
S88A
K90A
E94A
L97A
E102A
P108A
Q110A
Y112A
T117A
I119A
K121A
K129A
I132A
Q133A
Strep-DX2
GFP-K4B
IP:Strep
WCL
kDa
53
25
42
GFP
Strep
Actin
Relative binding
1.2
1.0
0.8
0.6
0.4
0.2
0
DX2

o

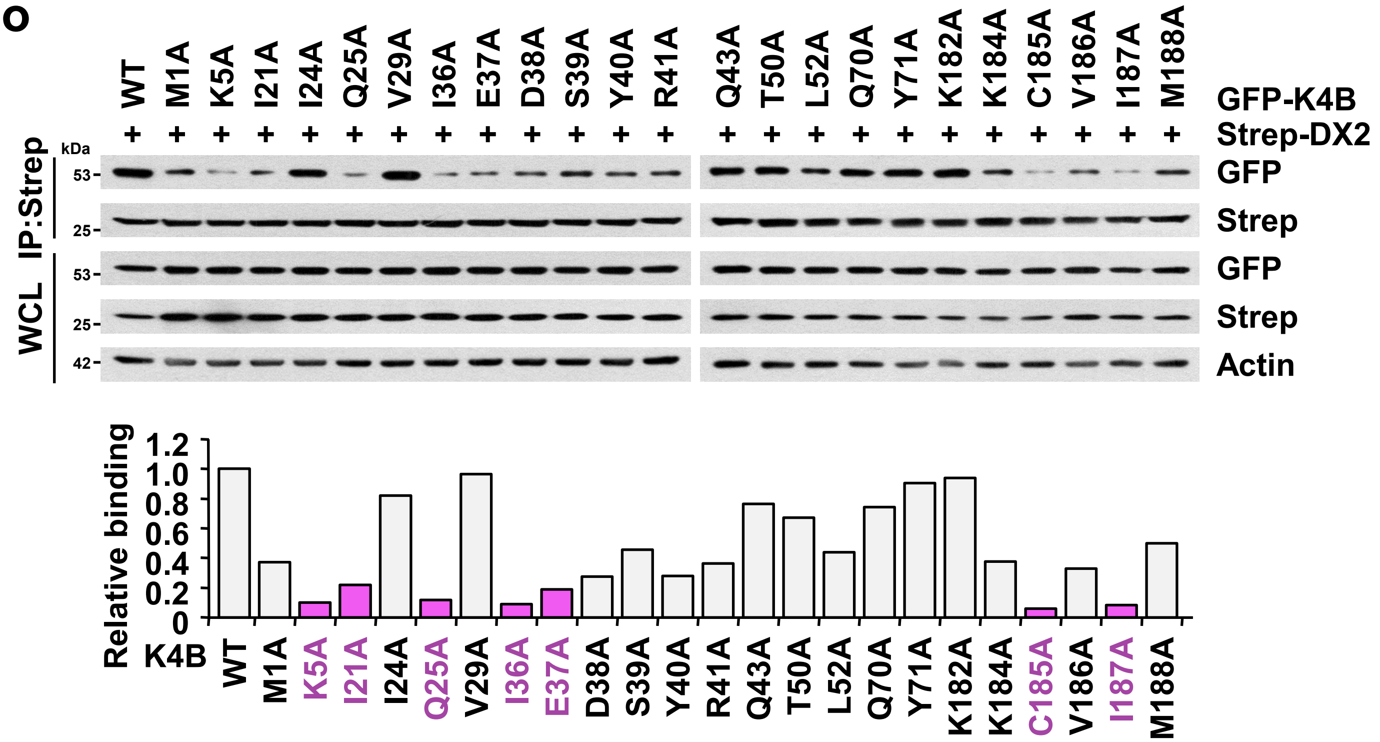
WT
M1A
K5A
I21A
I24A
Q25A
V29A
I36A
E37A
D38A
S39A
Y40A
R41A
Q43A
T50A
L52A
Q70A
Y71A
K182A
K184A
C185A
V186A
I187A
M188A
GFP-K4B
Strep-DX2
IP:Strep
WCL
kDa
53
25
42
GFP
Strep
Actin
Relative binding
1.2
1.0
0.8
0.6
0.4
0.2
0
K4B

Supplementary Fig. 5

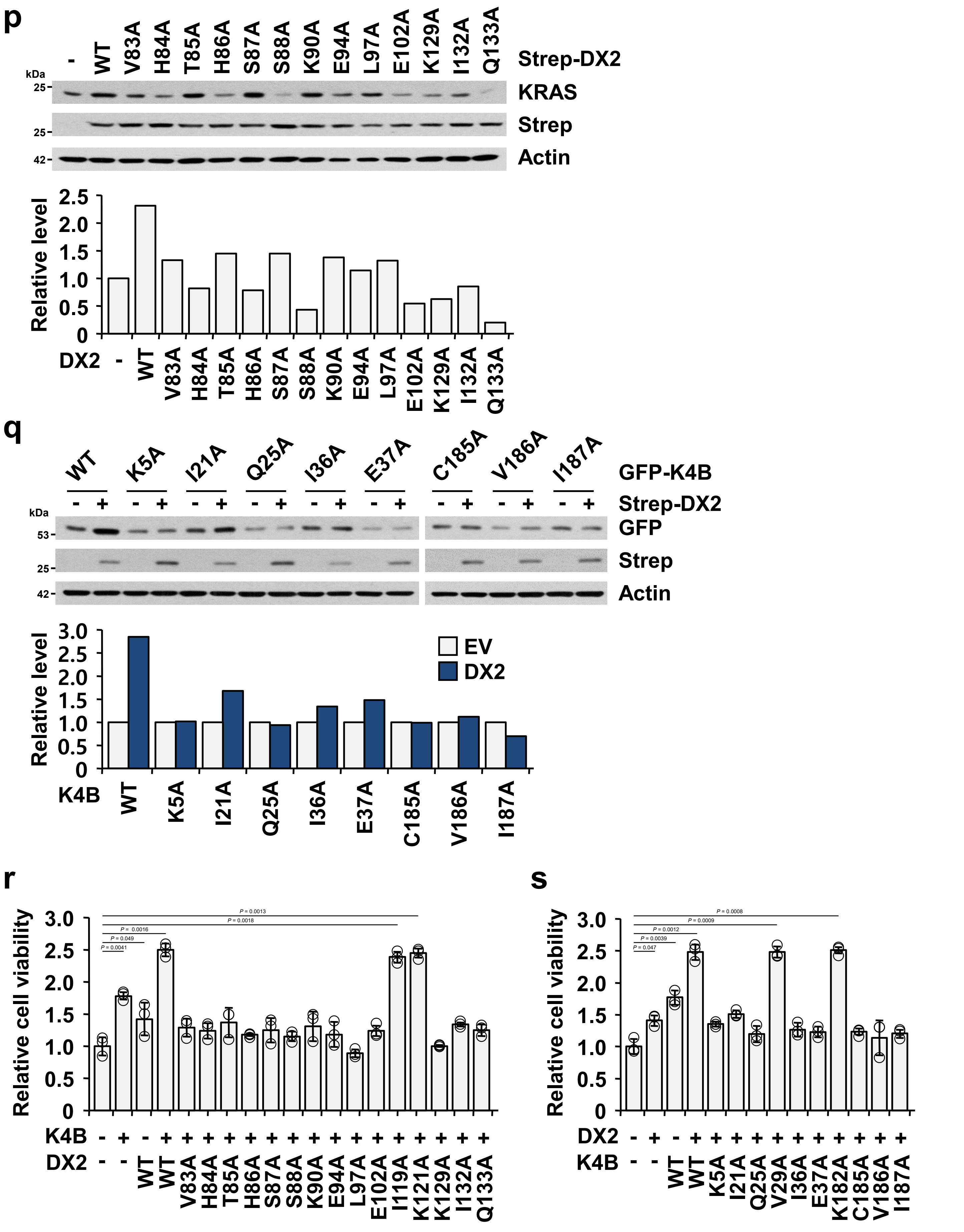

Supplementary Fig. 5 Determination of the binding mode of DX2 with KRAS. a Strep-DX2 mutants were introduced into 293T cells expressing nanoluciferase-KRAS4B and precipitated by the strep-tactin column. The amounts of co-precipitated KRAS4B were measured by the luciferase assay and presented as a bar graph. Experiments were independently repeated thrice and error bars denote the S.D. All error bars represent the standard deviation (S.D.). **b** Mapping results from chemical shift perturbation of ^{15}N-labeled $DX2_{51\text{-}251}$(C136S, C222S) by binding to KRAS4B HVR peptide (Fig. 3a). The residues showing strong ($\Delta\delta > 0.02$ ppm) and medium ($0.01 < CSP \leq 0.02$ ppm) perturbation are shown in red and orange, respectively, on the surface of DX2 (left). The weighted average of chemical shift changes, Δδ, was calculated by the equation $\Delta\delta \text{ (ppm)} = (\delta H^2 + 0.2 \times \delta N^2)^{1/2}$. The surface electrostatic potential of DX2 was presented in the same orientation (right). **c** ^{1}H-^{15}N TROSY spectra of ^{15}N-labeled $DX2_{51\text{-}251}$(C136S, C222S) (left) in the presence of HRAS (middle) or RALA HVR peptide (right). **d** Root mean square deviation (RMSD) of Cα atoms of the DX2-KRAS4B complex from nine MD simulation trajectories. Among the three trials at three time points, one selected main trajectory was extended to 600 ns (black). **e** Cα-RMSD of DX2 (green), KRAS4B (violet), and DX2-KRAS4B complex (black) structure over simulation time for the main trajectory. **f** Distance of Cα atoms between DX2 H86 and KRAS4B I187. **g** Cα-RMSD of KRAS4B (violet), HVR (magenta), and G domain (orange). For the Cα-RMSD calculations, the initial frame was used as reference structure and the RMSD fitted by itself. **h** Result of the MD simulation to determine the binding surface of DX2 and KRAS4B. The proteins were presented as a surface model and two significant interfaces are highlighted by the dashed box (Fig. 3c). **i**, **j** Distance of hydrogen bond (**i**) and Pi interactions and salt bridges (**j**) of the indicated key residue pairs calculated from the main trajectory of the DX2-KRAS4B system. **k** Final snapshot of KRAS4B (magenta) superimposed with its GTP-bound conformation (yellow). The binding residues are presented by a stick. **l**, **m** Contact surface area of the binding residues in DX2 (**l**) and KRAS4B (**m**) from the representative snapshot at 533.7 ns. **n**, **o** Validation of the residues implicated as critical for the binding between DX2 and KRAS4B. The 293T cells expressing Strep-DX2 (**n**) or -KRAS4B (**o**) mutants were subjected to immunoprecipitation using the anti-strep antibody. Precipitates were separated by SDS-PAGE and the binding of two proteins was determined by immunoblotting using their specific antibodies. The results were quantified and important residues of DX2 and KRAS4B were highlighted in light green and magenta colors, respectively, in the graph below each experiment. **p**-**s** Characterization of DX2-mediated cellular level and activity of KRAS using the binding-defective mutants of two proteins. DX2-dependent increase in the KRAS levels in cells expressing the indicated mutants of the two proteins was detected by immunoblotting using specific antibodies. The results were quantified and presented as a bar graph below each experiment (**p**, **q**). Same cells as above were subjected to MTT assay for checking KRAS4B-mediated control of cell viability. (**r**, **s**). **r**, **s** All the experiments were independently repeated thrice and error bars denote the S.D. Data are presented as mean values +/- S.D. *P*-value is from the two-sided t-test. Source data are provided as a Source data file. **n-q** Results are representative of at least three independent experiments.

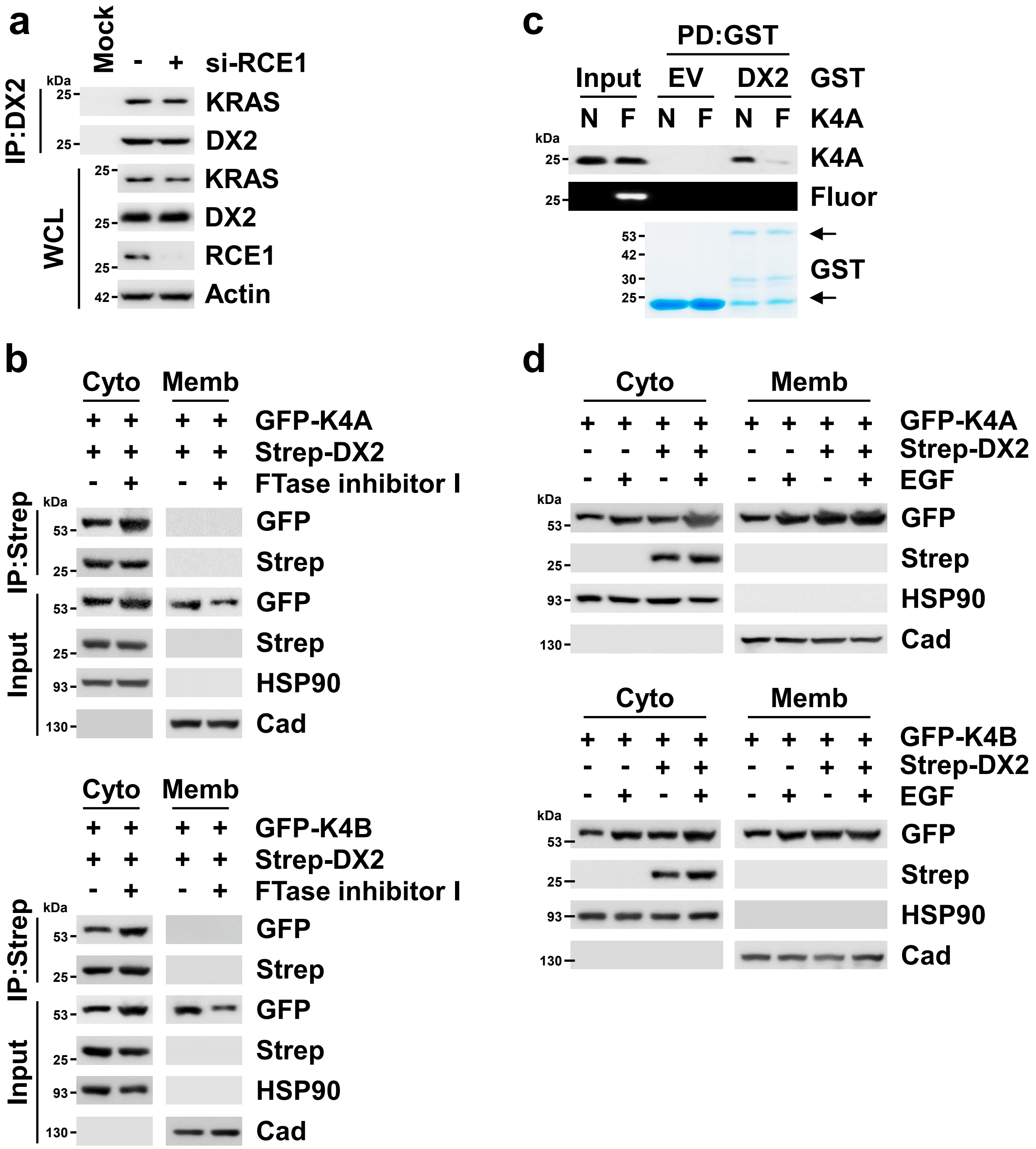

Supplementary Fig. 6 Determination of the KRAS status for DX2 binding. a Validation of RCE-1 implicated for the binding of KRAS and DX2. H460 cells expressing si-RCE1 were immunoprecipitated by using the anti-DX2 antibody. Precipitates were separated and detected by SDS-PAGE and immunoblotting, respectively. **b** The 293T cells expressing KRAS4A (upper) or KRAS4B (bottom) with DX2 were treated with FTase inhibitor I and separated to cytosol (Cyto) and membrane (Memb) fractions. HSP90 and cadherin (Cad) were used as loading markers for the cytosol and membrane, respectively. **c** *In vitro* pull-down assay to determine the DX2 binding to farnesylated KRAS4A. *In vitro* farnestlyated KRAS4A was incubated with purified GST-DX2. Binding of the two proteins and KRAS farnesylation were determined by immunoblotting and fluorescent scanning, respectively. N and F mean native and farnesylated KRAS4A, respectively. **d** The 293T cells expressing GFP-KRAS4A (upper) or 4B (bottom) with Strep-DX2 were treated with EGF and separated into cytosol and membrane fractions. Source data are provided as a Source data file. **a-d** Results are representative of at least three independent experiments.

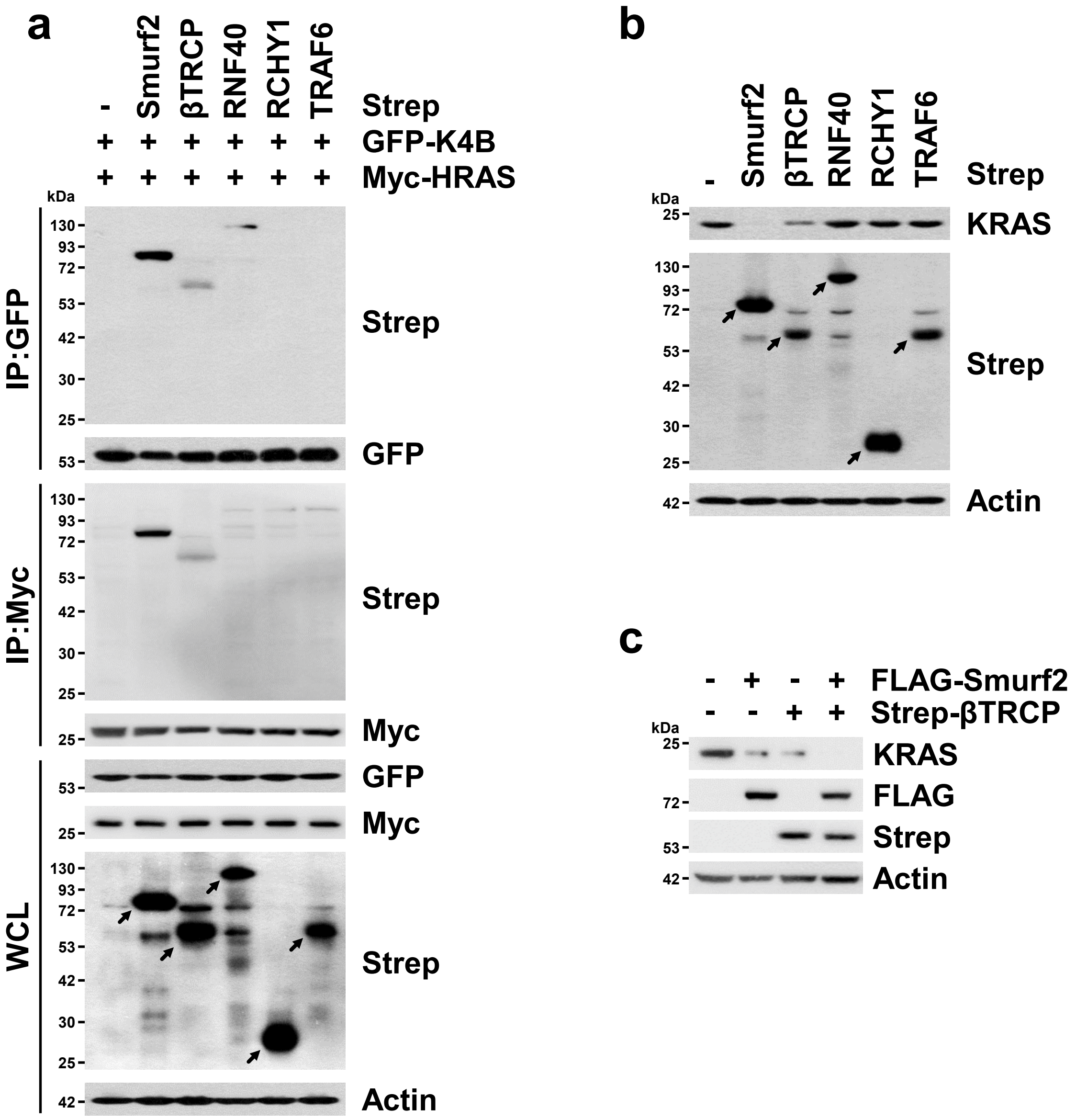

Supplementary Fig. 7 Identification of specific E3 ligase, Smruf2, against KRAS. a, **b** Determination of the E3 ligase responsible for ubiquitination of KRAS4B. The 293T cells expressing GFP-KRAS4B and Myc-HRAS were introduced into Strep-Smurf2, βTRCP, RNF40, RCHY1, and TRAF6, and treated with MG-132 for 12 h. KRAS4B or HRAS was immunoprecipitated with the anti-GFP or -Myc antibody and co-precipitated proteins were detected by SDS-PAGE and immunoblotting using the anti-strep antibody (**a**). Cellular levels of endogenous KRAS were monitored in 293T cells expressing the above E3 ligases (**b**). **c** H460 cells expressing FLAG-Smurf2 and Strep-βTRCP were treated with EGF and subjected to SDS-PAGE and immunoblotting. Source data are provided as a Source data file. **a-c** Results are representative of at least three independent experiments.

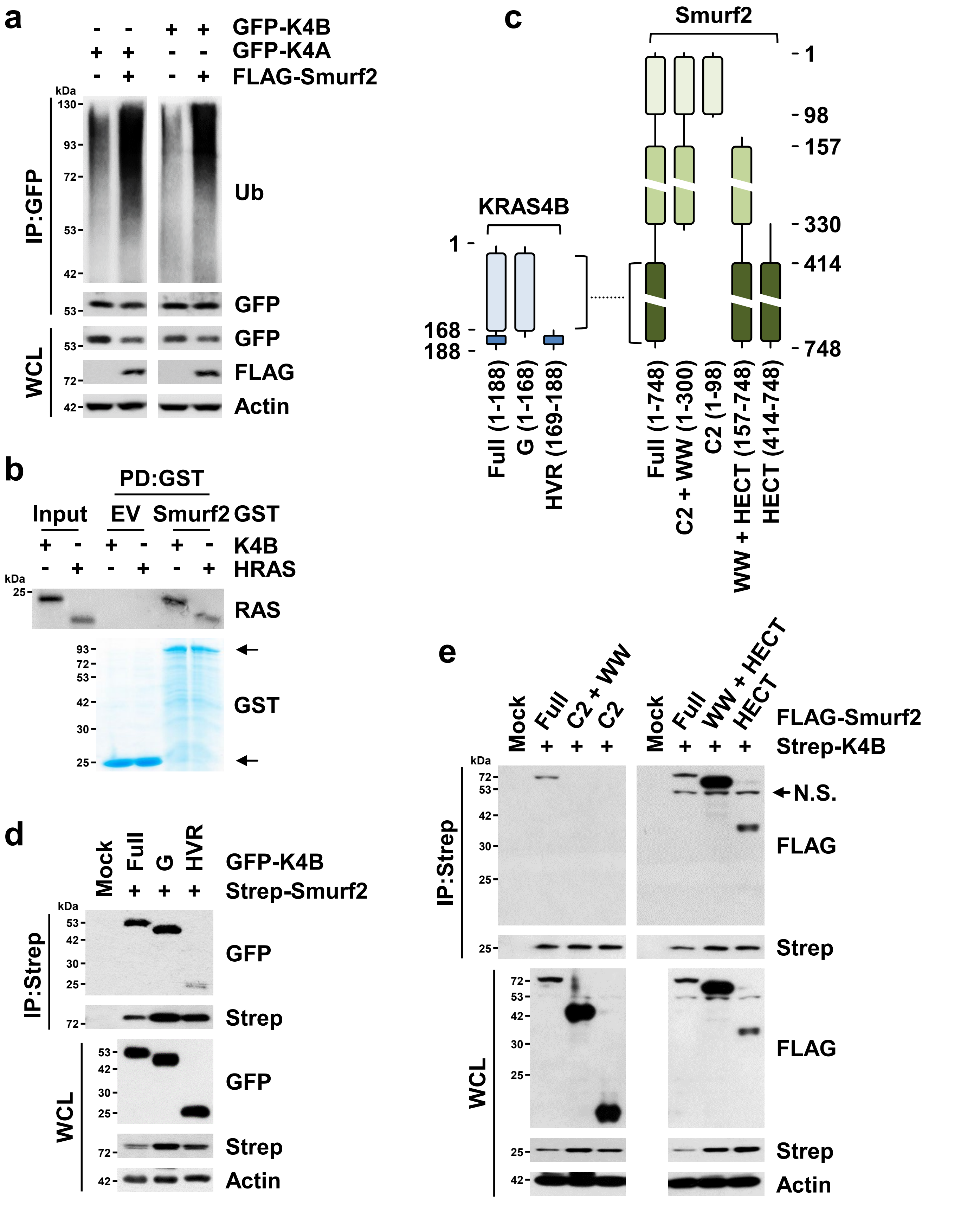
a
GFP-K4B
GFP-K4A
FLAG-Smurf2
IP:GFP
WCL
Ub
GFP
FLAG
Actin
b
PD:GST
Input
EV
Smurf2
GST
K4B
HRAS
RAS
c
KRAS4B
Smurf2
Full (1-188)
G (1-168)
HVR (169-188)
Full (1-748)
C2 + WW (1-300)
C2 (1-98)
WW + HECT (157-748)
HECT (414-748)
d
Mock
Full
G
HVR
GFP-K4B
Strep-Smurf2
IP:Strep
WCL
GFP
Strep
Actin
e
C2 + WW
WW + HECT
HECT
FLAG-Smurf2
Strep-K4B
N.S.
FLAG
Strep
Actin

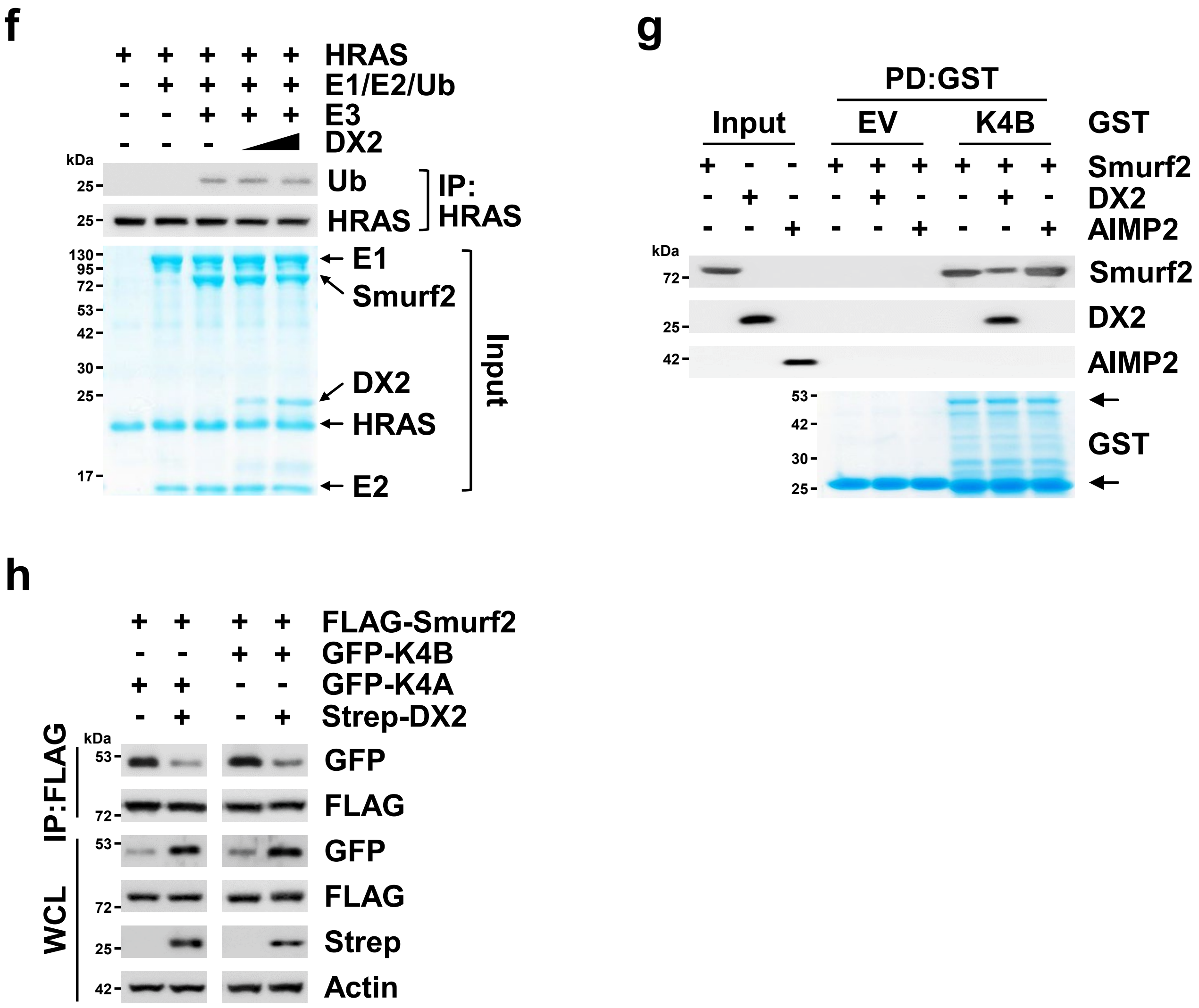
f
HRAS
E1/E2/Ub
E3
DX2
kDa
Ub
HRAS
IP: HRAS
E1
Smurf2
DX2
HRAS
E2
Input
g
PD:GST
Input
EV
K4B
GST
Smurf2
DX2
AIMP2
kDa
Smurf2
DX2
AIMP2
GST
h
FLAG-Smurf2
GFP-K4B
GFP-K4A
Strep-DX2
kDa
IP:FLAG
WCL
GFP
FLAG
GFP
FLAG
Strep
Actin

Supplementary Fig. 8 Smurf2-mediated ubiquitination of KRAS. a The 293T cells expressing GFP-KRAS4A, 4B, and FLAG-Smurf2 in the indicated combinations were treated with MG-132 for 24 h and subjected to immunoprecipitation with the anti-GFP antibody. Amounts of the ubiquitinated RAS were determined by immunoblotting using the anti-ub antibody. **b** *In vitro* pull-down assay for the binding of Smurf2 to KRAS4B or HRAS. Purified KRAS4B or HRAS were mixed with GST-Smurf2 proteins and co-precipitates with GST-tagged proteins were determined by immunoblotting with the pan-RAS antibody and Coomassie staining, respectively. **c**-**e** Determination of domains responsible for Smurf2-KRAS4B binding. Domain arrangements of Smurf2 and KRAS4B. The domains involved in the binding of the two proteins are marked with brackets connected by dashed line (**c**). The 293T cells expressing Strep-Smurf2 were introduced with each domain of GFP-KRAS4B and immunoprecipitated with the anti-Strep antibody. Co-precipitates were analyzed by immunoblotting using their specific antibodies (**d**). The 293T cells co-expressing each domain of FLAG-Smruf2 and Strep-KRAS4B were subjected to immunoprecipitation. Co-precipitated Smurf2 domain with KRAS4B was monitored as above. N.S. denotes non-specific blotted signal (**e**). **f** *In vitro* ubiquitination assay analyzing the effect of DX2 on Smurf2-mediated ubiquitination of HRAS. UBE1, UbcH5c/UBE2D3, Smurf2, and HRAS were used as E1, E2, E3, and substrate for the reaction, respectively. The ubiquitinated amounts of HRAS were detected by immunoblotting (upper) and proteins used in the reaction were confirmed by Coomassie staining in input panel (bottom). **g** *In vitro* pull-down assay determining the inhibitory effect of DX2 or AIMP2 on the interaction between KRAS4B and Smurf2. Smurf2 and GST-KRAS4B proteins were mixed and then DX2 or AIMP2 added. Precipitated proteins with GST-KRAS4B were detected by immunoblotting and Coomassie staining. **h** The 293T cells expressing FLAG-Smurf2, Strep-DX2, and GFP-KRAS4A and 4B in the indicated combinations were subjected to immunoprecipitation using the anti-FLAG antibody. The proteins co-precipitated with Smurf2 were determined by immunoblotting with the specific antibodies. Source data are provided as a Source data file. **a**-**b**, **d**-**h** Results are representative of at least three independent experiments.

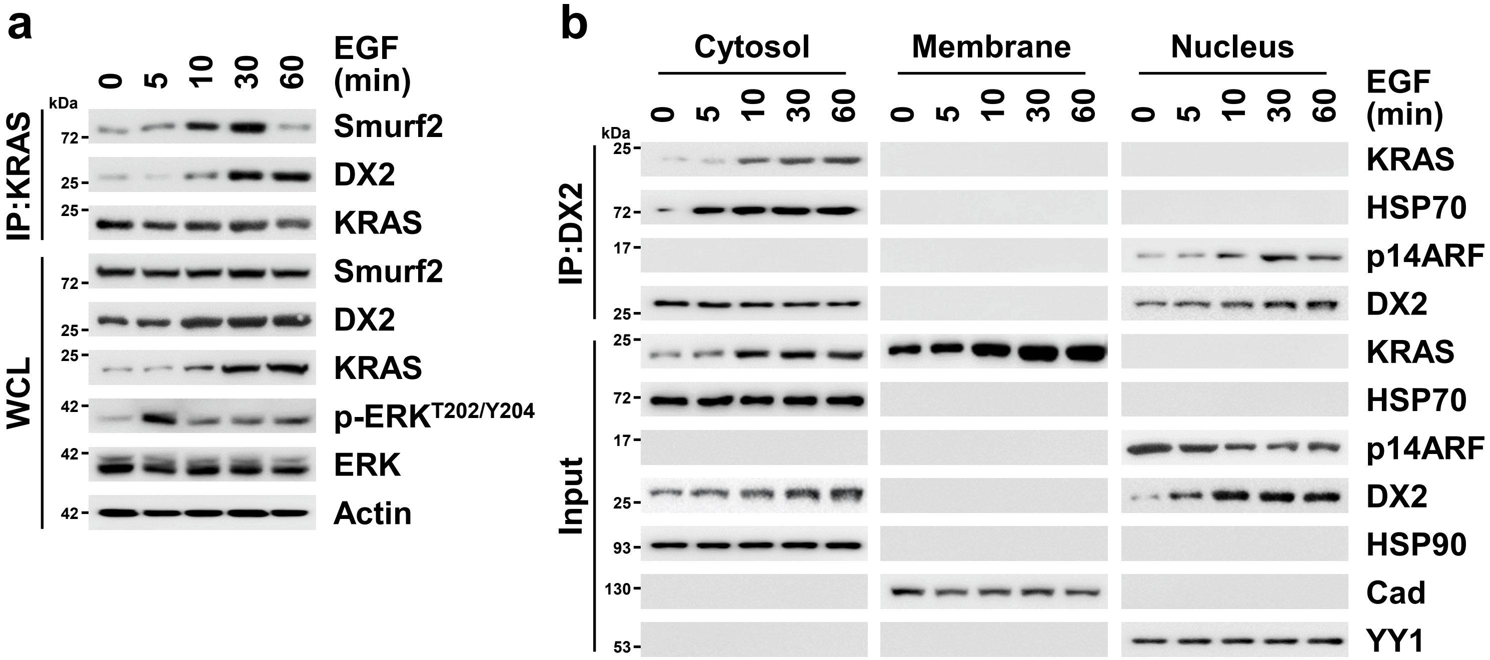

Supplementary Fig. 9 Analysis of EGF-dependent binding kinetics of KRAS and DX2. a, **b** H460 cells treated with EGF in a time-dependent manner were subjected to immunoprecipitation using the anti-KRAS antibody (**a**). Same cells were separated into the cytosol, membrane, and nucleus fractions, and DX2 in each fraction was immunoprecipitated by its specific antibody (**b**). All the binding results were quantified and presented as a graph (Fig. 4g). Source data are provided as a Source data file. **a-b** Results are representative of at least three independent experiments.

a

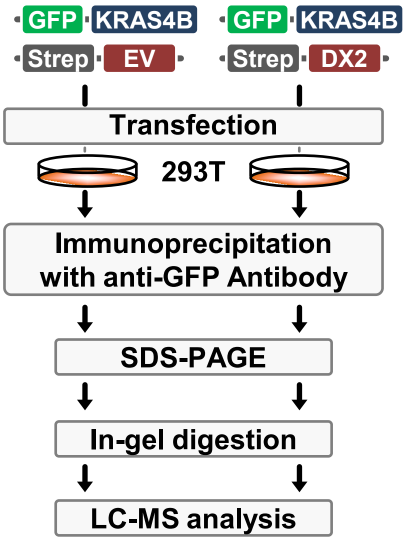

**DX2 -**	
**Peptide**	**Residue**
^{102}RVkDSEDVPMVLVGNk^{117}	K104, K117
^{89}SFEDIHHYREQIkR^{102}	K101
^{105}DSEDVPMVLVGNkCDLPSR^{123}	K117
^{179}kkSk^{182}	K179, K180, K182
^{74}TGEGFLCVFAINNTk^{88}	K88
^{102}RVkDSEDVPMVLVGNK^{117}	K104
^{104}VkDSEDVPMVLVGNK^{118}	K105
**DX2 +**	
**Peptide**	**Residue**
^{89}SFEDIHHYREQIkR^{102}	K101
^{103}VkDSEDVPMVLVGNK^{117}	K104
^{74}TGEGFLCVFAINNTk^{88}	K88
^{102}RVkDSEDVPMVLVGNk^{117}	K104, K117

b

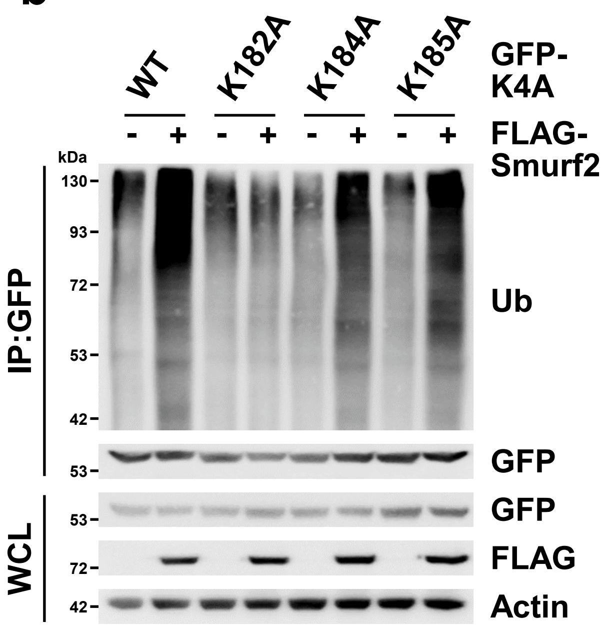

Supplementary Fig. 10 Identification of the ubiquitination sites of KRAS. a Strategy of mass spectrometry for identification of KRAS4B ubiquitination site to be affected by DX2. GFP-KRAS4B and Strep-DX2 were ectopically introduced into 293T cells and the cell extracts were subjected to immunoprecipitation with the antibody against GFP. Precipitates were subjected to SDS-PAGE and in-gel digestion for LC-MS analysis (left). Summarized table of the identified peptides containing ubiquitination sites in the sample without or with DX2 (right). Ubiquitinated lysine residues are denoted by red. **b** Identification of the Smurf2-mediated KRAS4A ubiquitination site using alanine substitution mutants. The 293T cells expressing GFP-KRAS4A mutants and FLAG-Smurf2 were treated with MG-132 and precipitated by the anti-GFP antibody. The ubiquitinated amounts of KRAS4A were monitored by immunoblotting using the anti-ub antibody. Source data are provided as a Source data file. Results are representative of at least three independent experiments.

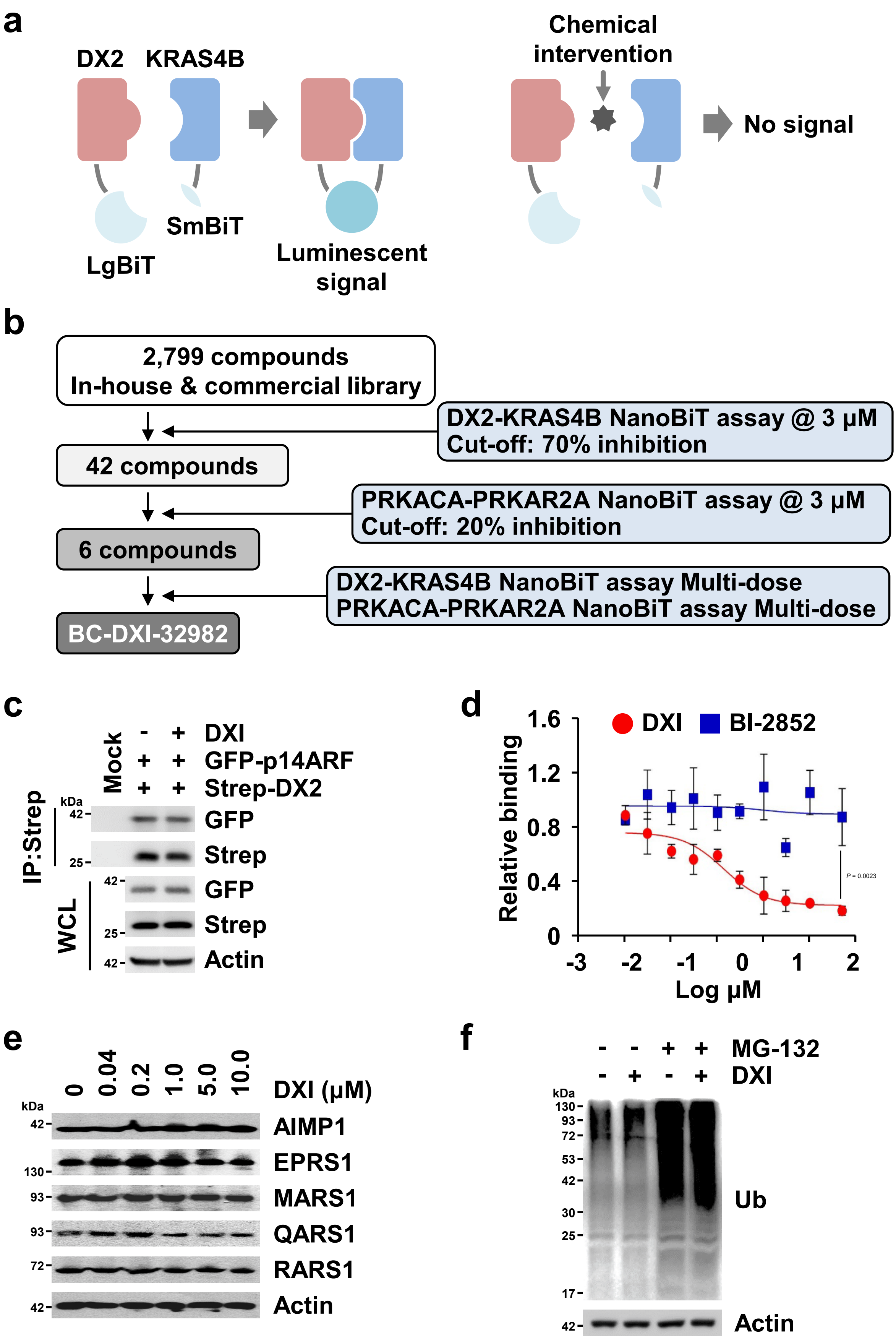
a
DX2
KRAS4B
LgBiT
SmBiT
Luminescent signal
Chemical intervention
No signal
b
2,799 compounds
In-house & commercial library
DX2-KRAS4B NanoBiT assay @ 3 μM
Cut-off: 70% inhibition
42 compounds
PRKACA-PRKAR2A NanoBiT assay @ 3 μM
Cut-off: 20% inhibition
6 compounds
DX2-KRAS4B NanoBiT assay Multi-dose
PRKACA-PRKAR2A NanoBiT assay Multi-dose
BC-DXI-32982
c
Mock
DXI
GFP-p14ARF
Strep-DX2
IP:Strep
WCL
GFP
Strep
Actin
d
DXI
BI-2852
Relative binding
Log μM
P = 0.0023
e
DXI (μM)
AIMP1
EPRS1
MARS1
QARS1
RARS1
Actin
f
MG-132
DXI
Ub
Actin

Supplementary Fig. 11 Screening of the DX2-KRAS interaction inhibitor. a Schematic representation of the screening assay using a NanoBiT system for identifying the chemicals that inhibit the interaction between DX2 and KRAS4B. The luminescent signal in the CHO-K1 cells expressing LgBiT-DX2 and SmBiT-KRAS4B proteins is abolished by chemical inhibition of the DX2-KRAS4B interaction. **b** Flow chart of the screening strategy. The chemicals (2,799) collected from the in-house and commercial chemical library were primarily screened at 3 μM *via* assay as described above and 42 chemicals with over 70% inhibition were subjected to counter screening. PRKACA and PRKAR2A were used as the negative controls to check the target specificity and 6 chemicals were identified as hit compounds. Among the 6 chemicals, BC-DXI-32982 was selected for further study as it showed the strongest potency in the dose-dependency test. **c**, **d** Checking the target specificity of DXI in the binding between DX2 and KRAS. The 293T cells expressing GFP-p14ARF and Strep-DX2 were treated with DXI and subjected to immunoprecipitation using the anti-strep antibody (**c**). DXI or BI-2852, the reported KRAS catalytic inhibitor, was treated to CHO-K1 cells expressing LgBiT-DX2 and SmBiT-KRAS4B. The experiment was independently repeated three times and error bars denote the S.D. Data are presented as mean values +/- S.D. (**d**). **e** H460 cells treated with DXI in dose-dependent manner for 18 h were subjected to SDS-PAGE and immunoblotting (Fig. 5e). **f** H460 cells were treated with DXI and MG-132 and ubiquitination (Ub) of the whole proteins in the cell extracts was determined by immunoblotting with the anti-ub antibody. Source data are provided as a Source data file. **c**, **e**-**f** Results are representative of at least three independent experiments.

a

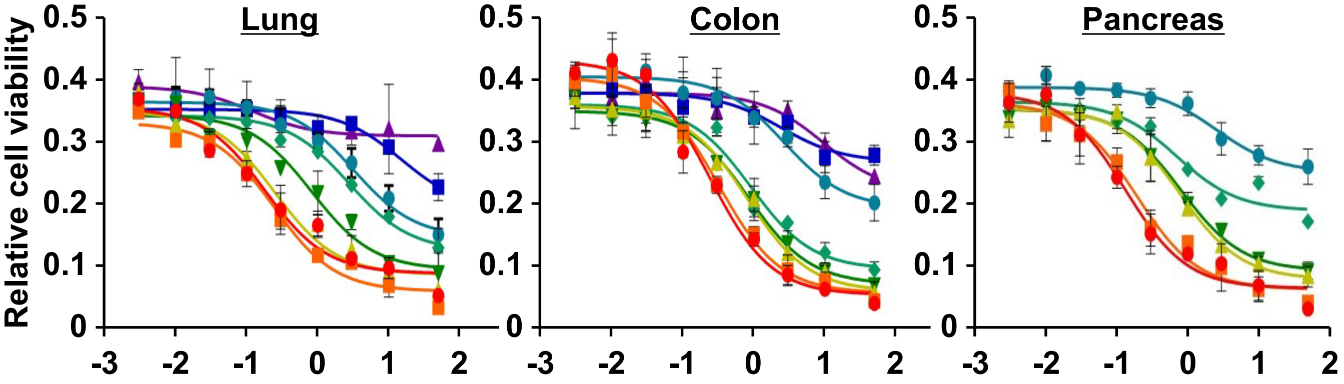

Lung	EC_{50} (M)
H460	$1.9 \pm 0.9 \times 10^{-7}$
HCC1588	$2.4 \pm 0.8 \times 10^{-7}$
H1650	$2.7 \pm 1.3 \times 10^{-7}$
A549	$8.8 \pm 4.6 \times 10^{-7}$
Calu-6	$2.8 \pm 0.8 \times 10^{-6}$
H1299	$4.0 \pm 3.5 \times 10^{-6}$
HCC44	N.D.
WI-26	N.D.

Colon	EC_{50} (M)
H747	$2.4 \pm 0.4 \times 10^{-7}$
SNU-407	$3.1 \pm 0.5 \times 10^{-7}$
DLD-1	$8.3 \pm 2.5 \times 10^{-7}$
LoVo	$8.5 \pm 1.3 \times 10^{-7}$
SNU-C4	$9.2 \pm 2.0 \times 10^{-7}$
HCT-8	$2.5 \pm 1.2 \times 10^{-6}$
COLO-205	N.D.
HCT-116	N.D.

Pancreas	EC_{50} (M)
CFPAC-1	$1.4 \pm 0.4 \times 10^{-7}$
MIA-PaCa2	$1.9 \pm 0.5 \times 10^{-7}$
Panc1	$8.3 \pm 4.0 \times 10^{-7}$
Panc10.05	$8.3 \pm 3.3 \times 10^{-7}$
AsPC-1	N.D.
SNU-410	N.D.

b

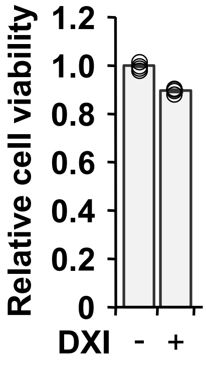

c

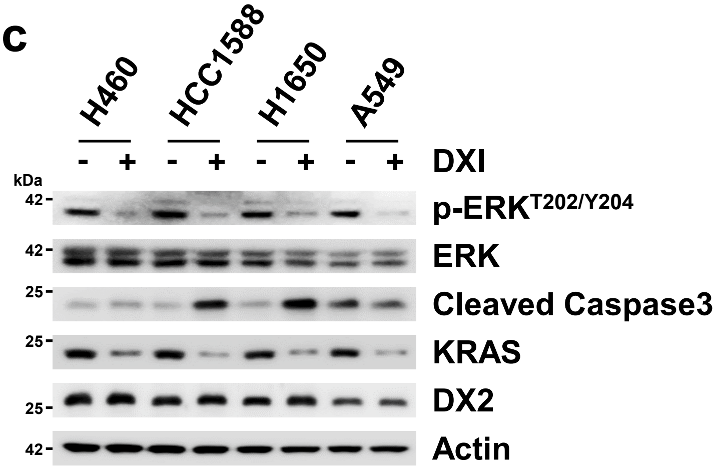

d

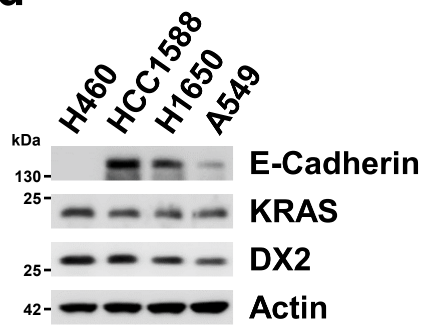

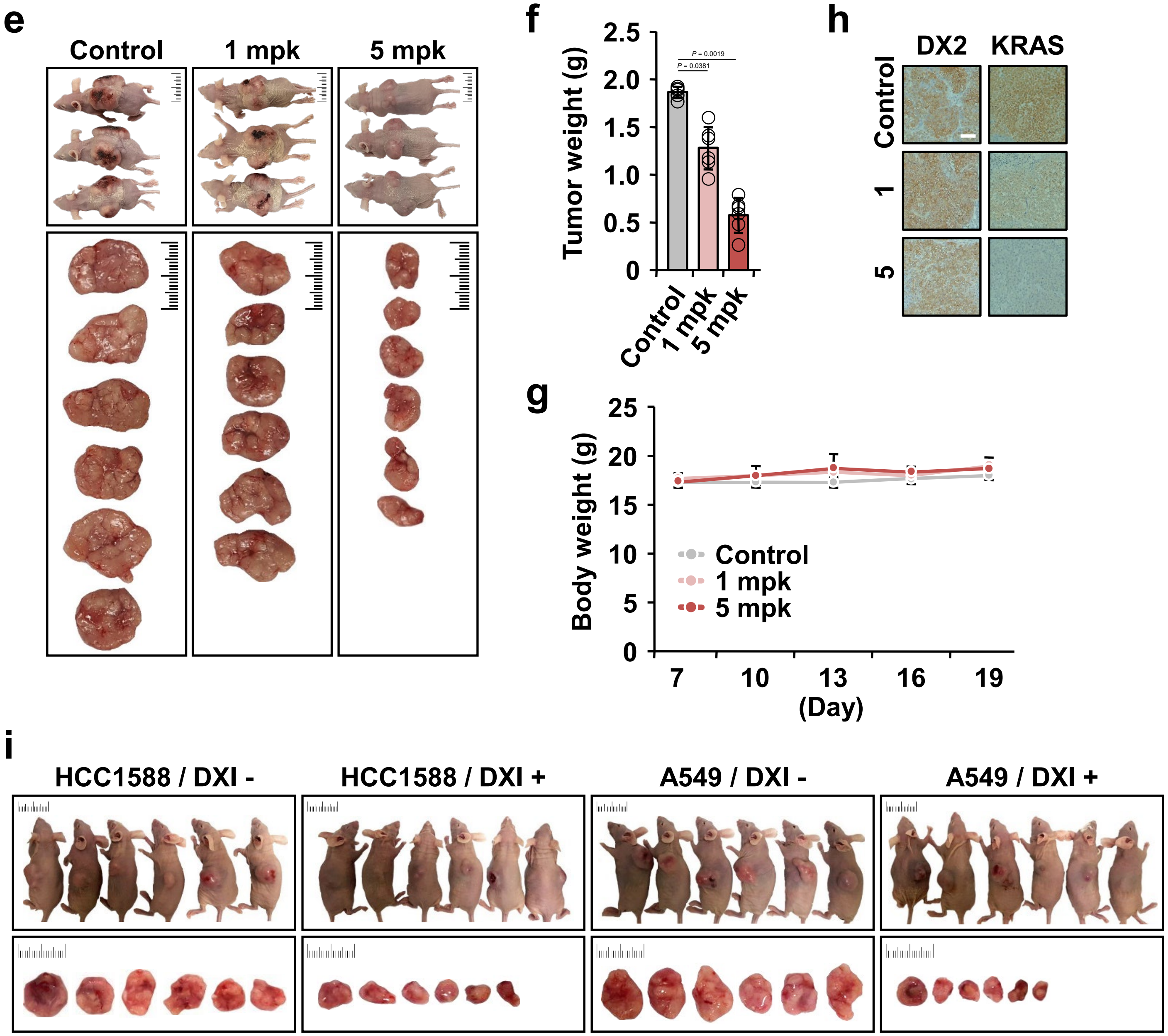

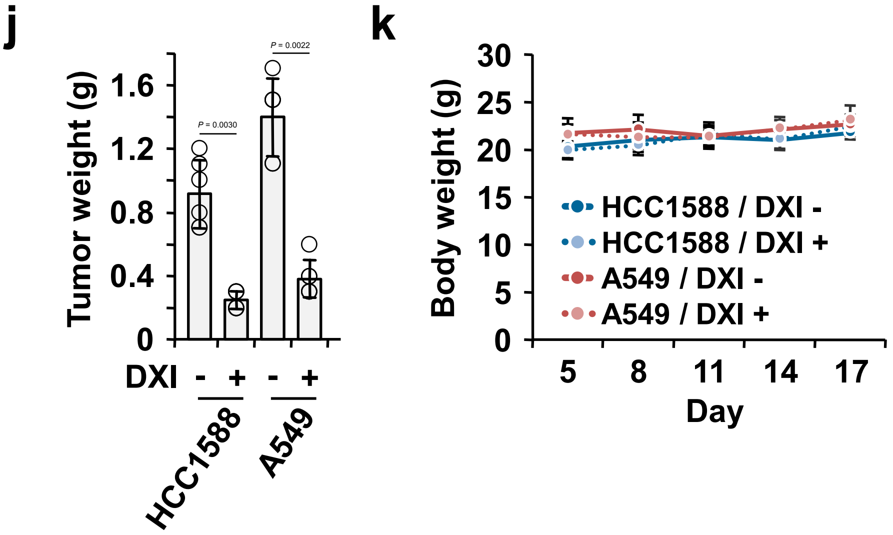

l

Concentration (ng/g)	
HCC1588	A549
23571±44221	8044±4524

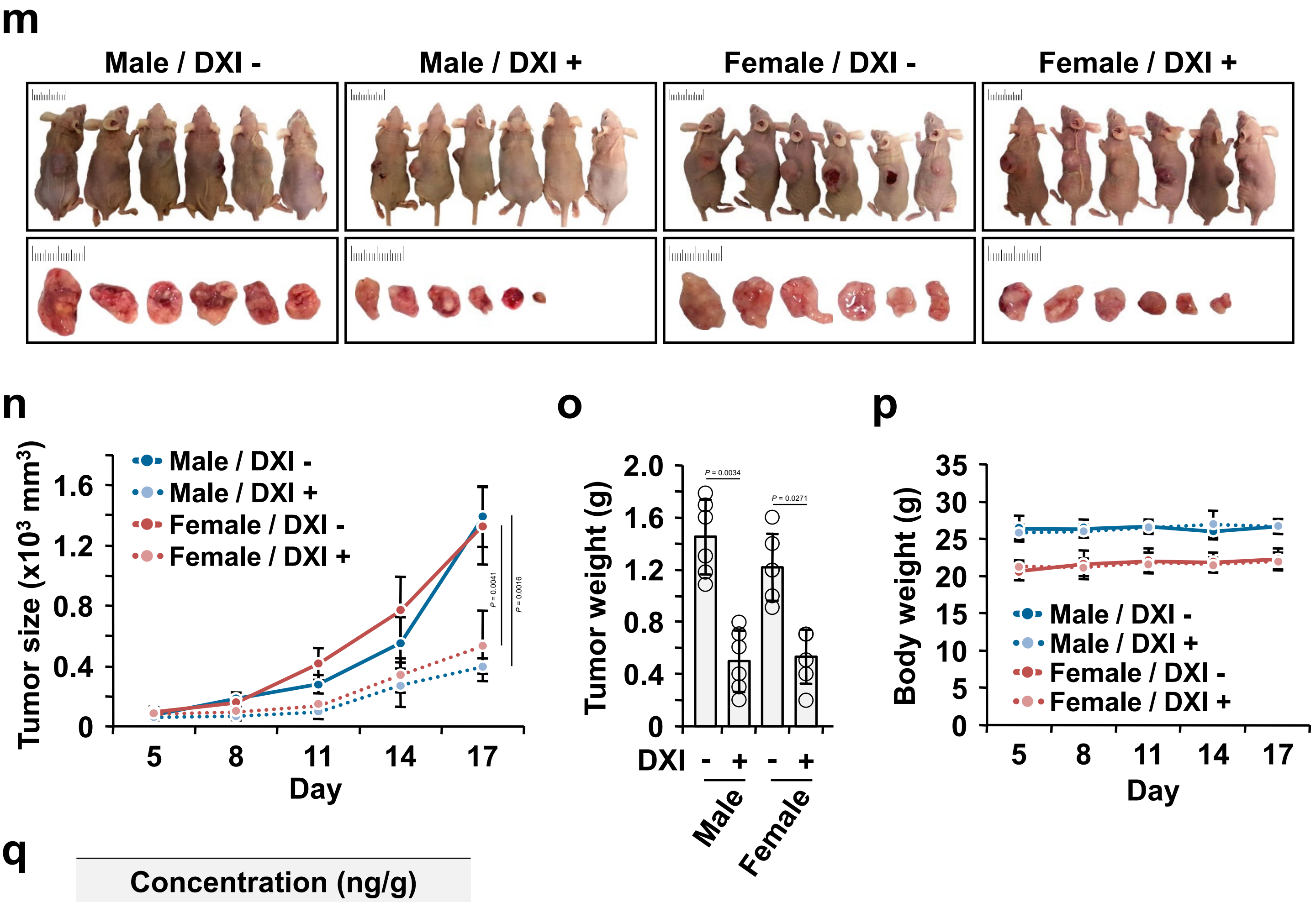

q

Concentration (ng/g)	
Male	Female
11242±4654	7917±3165

Supplementary Fig. 12 Suppression of the cell viability in a DX2-dependent manner *via* BC-DXI-32982. a Lung, colorectal, and pancreatic cell lines were treated with DXI in dose-dependent manner for 96 h and subjected to the MTT assay. The EC_{50} values of DXI for cell viability were calculated and are presented in the table (bottom). The EC_{50} value of each cell line is shown as a heat map (Fig. 5g). N.D. denotes "not determined". **b** DXI was treated to un-transformed MEF and cell viability was measured as described above. **c** H460, HCC1588, H1650, and A549 cells were treated with DXI and subjected to SDS-PAGE and immunoblotting using the indicated antibodies. **d** Indicated proteins in cell extracts from the above four cells were analyzed using their specific antibodies. **e**-**h**, *In vivo* effects of DXI on the tumor growth. Mice (n = 3) xenografted with H460 cells on the left and right backs were injected intraperitoneally with DXI (1 or 5 mg/kg) five times in 12 d. Images of the tumor-embedded mice (n = 3) and excised tumors (n = 6) are shown (**e**), along with the weight of the excised tumors (**f**). Body weights were measured for the stated experimental periods and are represented as a line graph (**g**). All error bars represent the standard deviation (S.D.). DX2 and KRAS levels in the excised tumors were analyzed by immunohistochemistry using their specific antibodies, and the images were obtained from different sites in the same tissue (scale bar = 400 μm). (**h**). **i**-**l** *In vivo* efficacy of DXI on the growth of tumors harboring the KRAS WT or mutation. HCC1588 or A549 cells were xenografted on one side back of mice (n = 6) and DXI was intravenously injected five times for 12 d. Images of tumor-bearing mice and the excised tumors were shown (**i**). The harvested tumor (**j**) and body weights (**k**) were measured. All error bars represent the standard deviation (S.D.). *P*-value is from the two-sided t-test. DXI levels in the excised tumors were analyzed and shown (**l**). **m**-**q**, Evaluation of DXI on sex as a biological variable (SABV). DXI was were intravenously injected into male and female mice (n = 6) xenografted with H460 cells five times for 12 d. Images were obtained as above (**m**). Tumor sizes (**n**) and body weights (**p**) were measured throughout the overall experimental periods. The excised tumors were also weighed (**o**). All error bars represent the standard deviation (S.D.). *P*-value is from the two-sided t-test. Chemical concentrations of the harvested tumors were determined (**q**). **a**, **b** All the experiments were independently repeated thrice and error bars denote the S.D. Data are presented as mean values +/- S.D. Source data are provided as a Source data file. **c**-**d**, **h** Results are representative of at least three independent experiments.

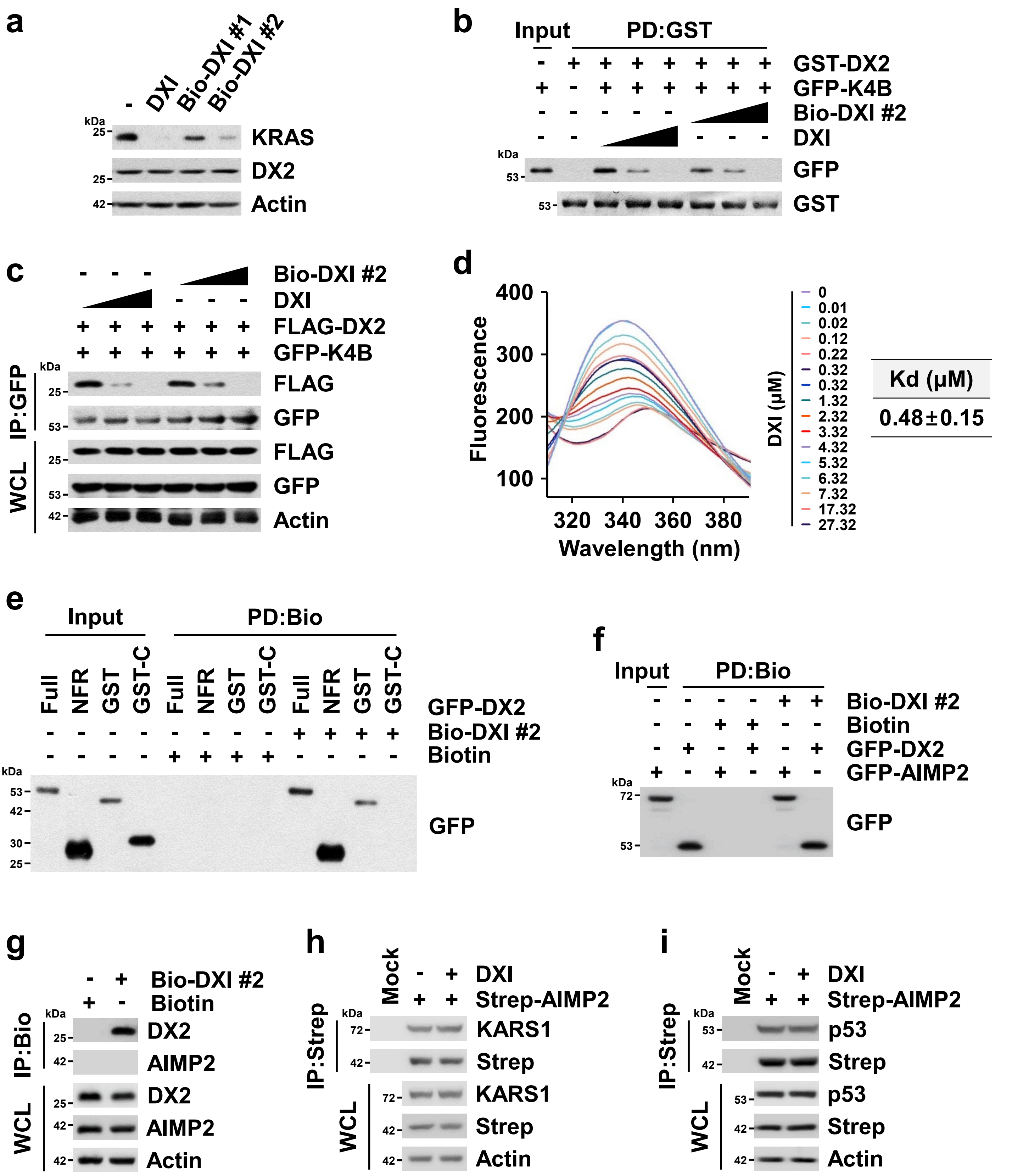
a
- DXI Bio-DXI #1 Bio-DXI #2
kDa
25
KRAS
25
DX2
42
Actin
b
Input
PD:GST
GST-DX2
GFP-K4B
Bio-DXI #2
DXI
kDa
53
GFP
53
GST
c
Bio-DXI #2
DXI
FLAG-DX2
GFP-K4B
kDa
IP:GFP
FLAG
GFP
WCL
FLAG
GFP
Actin
d
Fluorescence
400
300
200
100
320 340 360 380
Wavelength (nm)
DXI (μM)
0
0.01
0.02
0.12
0.22
0.32
0.32
1.32
2.32
3.32
4.32
5.32
6.32
7.32
17.32
27.32
Kd (μM)
0.48±0.15
e
Input
PD:Bio
Full NFR GST GST-C
GFP-DX2
Bio-DXI #2
Biotin
kDa
53
42
30
25
GFP
f
Input
PD:Bio
Bio-DXI #2
Biotin
GFP-DX2
GFP-AIMP2
kDa
72
53
GFP
g
Bio-DXI #2
Biotin
kDa
IP:Bio
DX2
AIMP2
WCL
DX2
AIMP2
Actin
h
Mock
DXI
Strep-AIMP2
kDa
IP:Strep
KARS1
Strep
WCL
KARS1
Strep
Actin
i
Mock
DXI
Strep-AIMP2
kDa
IP:Strep
p53
Strep
WCL
p53
Strep
Actin

Supplementary Fig. 13

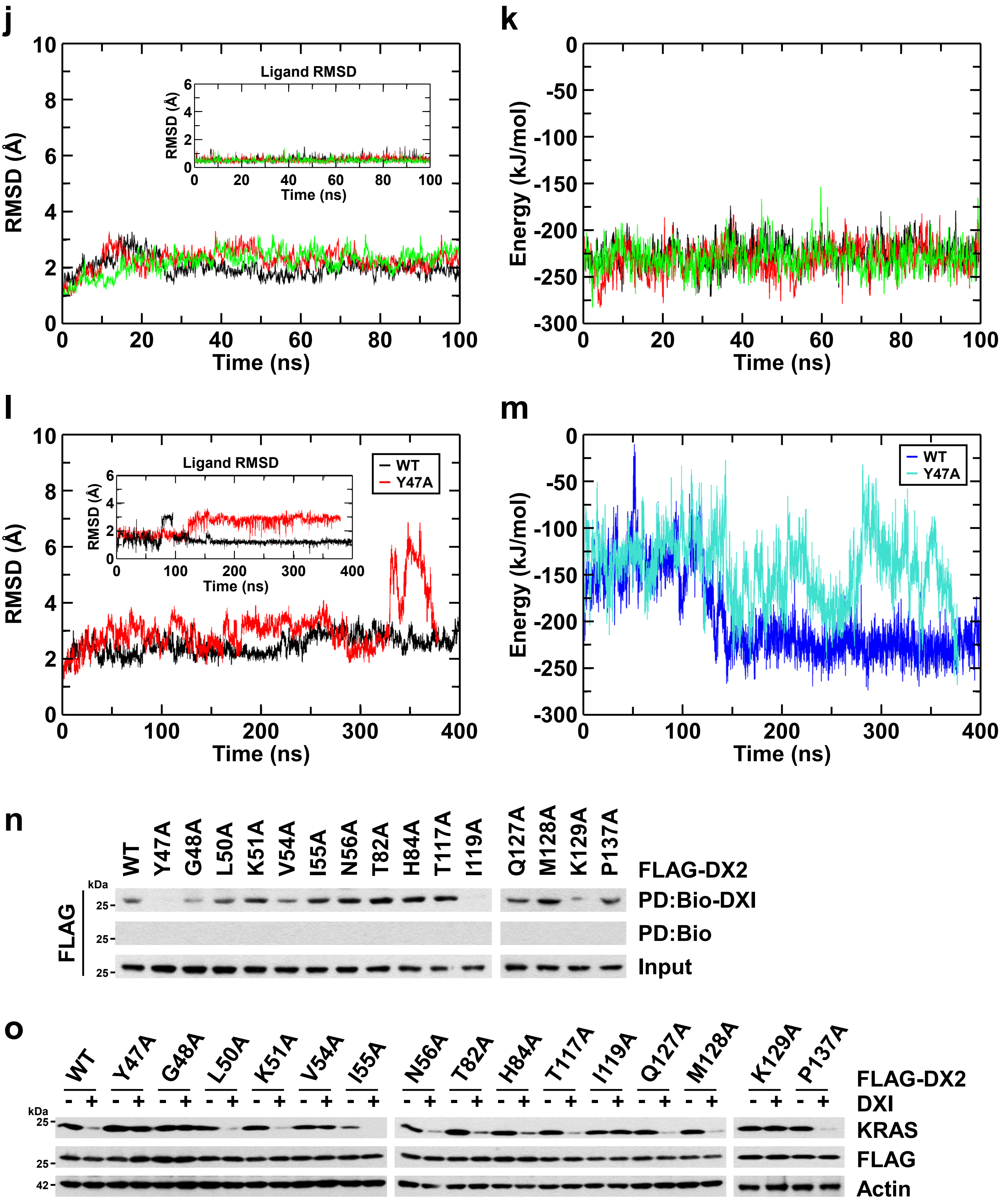

p

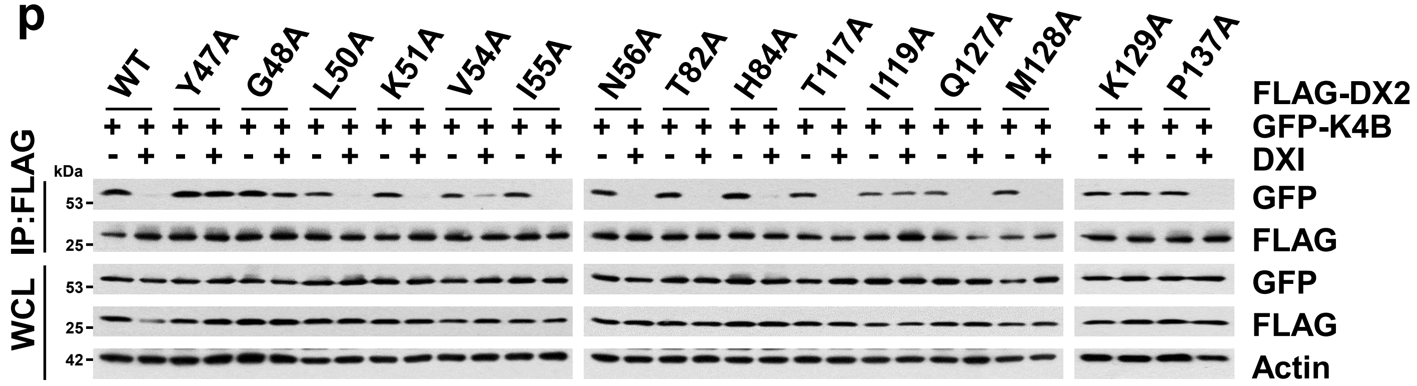
WT
Y47A
G48A
L50A
K51A
V54A
I55A
N56A
T82A
H84A
T117A
I119A
Q127A
M128A
K129A
P137A
FLAG-DX2
GFP-K4B
DXI
IP:FLAG
WCL
kDa
53
25
42
GFP
FLAG
Actin

q

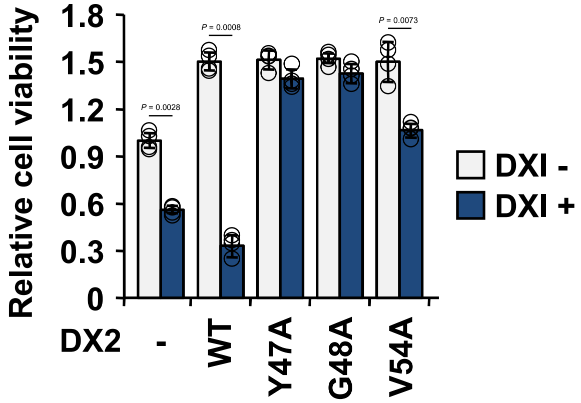
Relative cell viability
1.8
1.5
1.2
0.9
0.6
0.3
0
P = 0.0028
P = 0.0008
P = 0.0073
DX2
-
WT
Y47A
G48A
V54A
DXI -
DXI +

Supplementary Fig. 13 Determination of the suppressive mode of action of BC-DXI-32982 on the DX2-KRAS binding. a-**c** Validation of the effect of two biotin-conjugated DXIs (Bio-DXI #1 and #2) on DX2 levels (**a**), *in vitro* binding of DX2-KRAS4B (**b**), and cellular binding of two proteins (**c**). H460 cells were treated DXI or two other Biotin-DXIs (#1 and #2) and subjected to immunoblotting (**a**). Various concentration of Biotin-DXI #2 or DXI were added to a mixture of purified GST-DX2 protein and cell lysates containing GFP-KRAS4B. After precipitation with glutathione-Sepharose beads, precipitates were subjected to SDS-PAGE and immunoblotting. GST-DX2 proteins were detected by Coomassie staining (**b**). The 293T cells expressing FLAG-DX2 and GFP-KRAS4B were treated with Biotin-DXI #2 or DXI in a dose-dependent manner for 4 h and precipitated with the anti-GFP antibody (**c**). **d** The binding affinity of DXI to DX2 was determined by fluorescence-based binding titration measurements. The calculated Kd value is shown (n = 1). **e** Biotin-DXI #2 was mixed with the lysates including GFP-DX2 full length (Full), NFR, GST, and GST-C. Precipitates with streptavidin-Sepharose beads were subjected to SDS-PAGE and western blotting using the anti-GFP antibody. Biotin was used as a negative control for Biotin-DXI. **f** The cell lysates expressing GFP-DX2 or -AIMP2 were mixed with Biotin-DXI #2 and subjected to *in vitro* pull-down as described above. **g** H460 cells were treated with Biotin-DXI #2 and subjected to immunoprecipitation using streptavidin-Sepharose beads. **h** The 293T cells expressing Strep-AIMP2 were treated with DXI. Lysyl-tRNA synthetase 1 (KARS1) co-precipitated with AIMP2 was determined by SDS-PAGE and immunoblotting. **i** Binding of p53 to AIMP2 in the same cells as above was analyzed by immunoprecipitation. **j** Cα-RMSD of DX2 and ligand RMSD of DXI (inset) obtained from three replicate simulations of the DX2 and DXI complex started from the representative snapshot at 371.2 ns. **k** Non-bonded interaction energy of the DX2 and KRAS4B complex from the three replicates. **l** Cα-RMSD of DX2 and ligand RMSD of DXI (inset) for the WT (black) and Y47A (red) obtained from MD simulation of the DX2 and DXI complex. **m** Interaction energy of the three replicates between DX2 and KRAS4B. **n** The 293T cell lysates containing FLAG-DX2 mutants were mixed with Biotin-DXI and subjected to the *in vitro* pull-down assay. **o** H460 cells expressing FLAG-DX2 mutants were treated with DXI for 18 h and subjected to immunoblotting using the anti-KRAS and FLAG antibodies. **p** The 293T cells ectopically expressing the FLAG-DX2 mutants and GFP-KRAS4B were cultured in a medium with DXI for 4 h and immunoprecipitated using the anti-FLAG antibody. **q** A549 cells stably expressing the DX2 mutants were treated with DXI for 96 h. Cell viability was determined by the MTT assay and presented as a bar graph. Experiments were independently repeated thrice and error bars denote three S.D. Data are presented as mean values +/- S.D. *P*-value is from the two-sided t-test. Source data are provided as a Source data file. **a**-**c**, **e**-**i**, **n**-**p** Results are representative of at least three independent experiments.

Supplementary Fig. 14

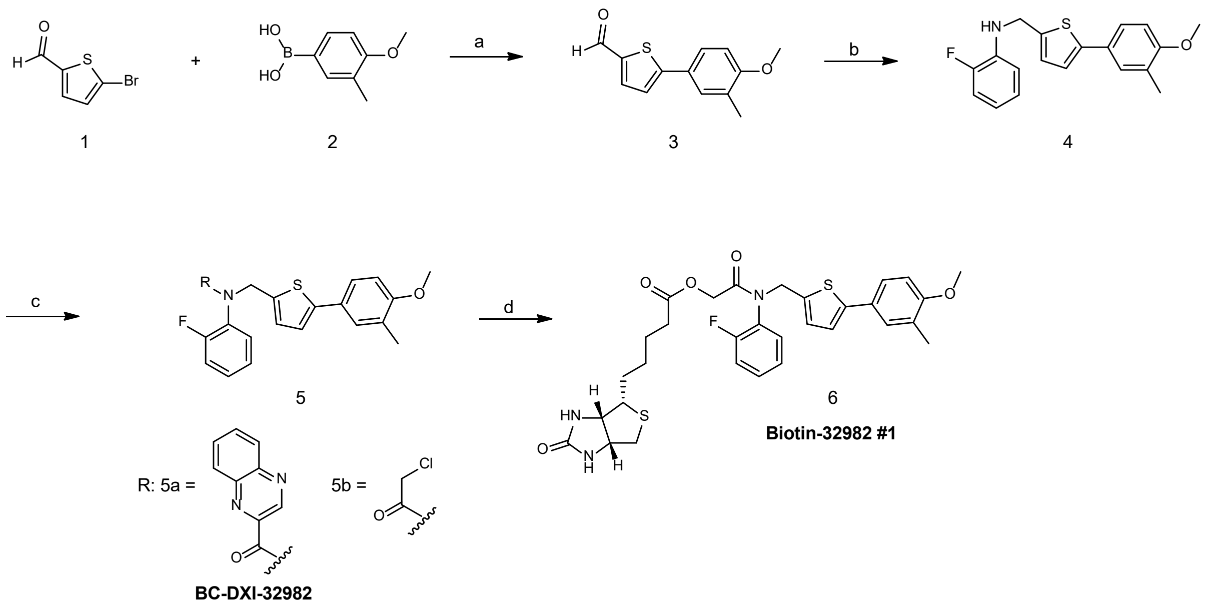

Supplementary Fig. 14 Scheme 1 for synthesis of BC-DXI-32982 and Biotin-32982 #1[a] [a]Reagents and conditions: (a) Pd(dppf)Cl_2.CH_2Cl_2 ([1,1′-Bis(diphenylphosphino)ferrocene]dichloropalladium(II), complex with dichloromethane), K_3PO_4 (Potassium phosphate), DMF(*N,N*-dimethylformamide):H_2O (4:1) mixture, 100 ºC, 2 h; (b) 2-fluoroaniline, cat (catalytic). acetic acid, CH_2Cl_2 (Dichloromethane), $NaCNBH_3$ (Sodium cyanoborohydride), MeOH (Methanol), 12 h; (c) 2-quinoxaloyl chloride for **5a** and chloroacetyl chloride for **5b**, TEA (Triethylamine), CH_2Cl_2, 0 ºC to rt (Room temperature), 12 h; (d) K_2CO_3 (Potassium carbonate), cat. KI (Potassium iodide), D-biotin, DMF, 12 h.

Supplementary Fig. 15

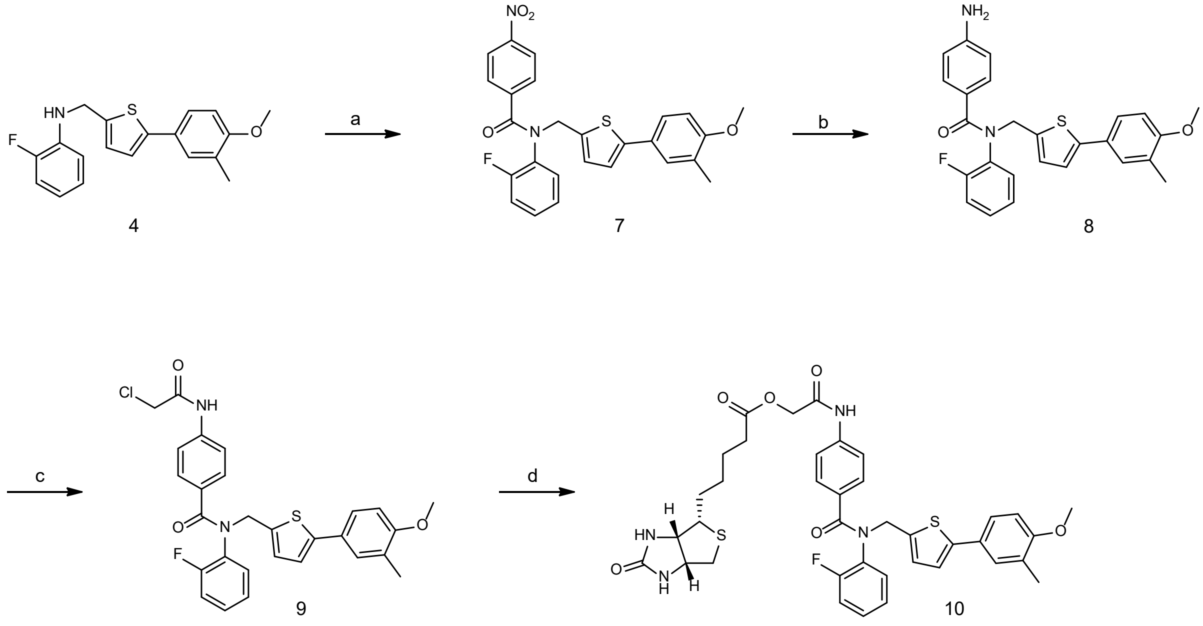

Biotin-32982 #2

Supplementary Fig. 15 Scheme 2 for synthesis of Biotin-32982 #2[a] [a]Reagents and conditions: (a) 4-nitrobenzoyl chloride, TEA, CH_2Cl_2, 0 ºC to rt, 12 h; (b) H_2, 10% Pd/C (Palladium on carbon), EtOAc (Ethyl acetate), 12 h; (c) chloroacetyl chloride, TEA, CH_2Cl_2, 0 ºC to rt, 12 h; (d) K_2CO_3, cat. KI, D-biotin, DMF, 12 h.

Supplementary Note 1

Pharmacokinetics of BC-DXI-32982

Male ICR mice (8 weeks old, 30~35 g) were acclimated to the testing facility in a temperature and humidity-controlled condition for approximately a week prior to the study. Dosing solutions made up to 1 mg/mL in dimethylacetamide/Tween 80/water (10/10/80 vol%) were dosed orally and by tail vein injection to the animals, respectively. The dosing solutions were administered via oral gavage at a dose of 10 mg/kg and a dose volume of 10 mL/kg or via tail vein injection at a dose of 5 mg/kg and a dose volume of 5 mL/kg, respectively. About 40μL of blood samples were collected into BD Microtainer plasma separator tubes at selected times through the saphenous vein over 24 hr post-dosing. Blood samples were centrifuged at 6,000 x g for 5 min to separate plasma and stored in a freezer until analyzed. Protein precipitation was conducted on 15 μL aliquot of plasma samples with 3 volumes of acetonitrile containing diclofenac as analytical internal standard and the mixture was vortexed. After centrifugation at 3,000xg for 30 min at 4°C, a 50 μL aliquot of supernatant was transferred to a 96-well plate and mixed with 50 μL of water. The resulting solutions were analyzed by Agilent 6460 QQQ LC-MS/MS system in a positive MRM mode.

Analytical method

1) Instrument: Agilent 6460 QQQ LC-MS/MS system equipped with dual AJS ESI ion source and Agilent 1200 series HPLC system

2) Data acquisition: positive MRM mode

BC-DXI-32982: m/z 506 > 217 (tR: 5.17 min); Diclofenac (IS): m/z 296 > 214 (tR: 4.61 min)

3) Column: Agilent Eclipse Plus C18, 3.5 μm, 2.1 x 100 mm

4) Mobile phase: linear gradient with 0.1% formic acid in de-ionized water (A) and 0.1% formic acid in acetonitrile (B)

Time (min)	%A	%B
0	95	5
2	95	5
3	5	95
8	5	95

5) Flow rate: 0.45 mL/min

6 Column temp.: 40oC

7) Injection vol.: 5 μL

Data analysis

The concentrations were determined from acquired MRM data using Agilent Mass Hunter Quantitative Analysis QQQ (ver. B.05.00). The chromatograms and full mass and MS/MS spectra were extracted using Qualitative Analysis Software.

Results

1) Plasma concentration of BC-DXI-32982 following a single IV and PO dose in mice

Time (hr)	Plasma concentration (ng/mL)	
	IV (5 mg/kg)	PO (10 mg/kg)

0.083	3463.1±1279.9	Missing
0.17	766.5±137.9	8.8±7.2
0.25	451.5±187.7	Missing
0.33	Missing	28.6±14.1
0.5	240.3±136.7	35.9±21.5
1	127.6±78.9	4.0±2.1
2	16.8±15.0	< 3.9
4	4.0	< 3.9
6	< 3.9	< 3.9
8	< 3.9	< 3.9
24	< 3.9	< 3.9

2) Plasma Pharmacokinetics of BC-DXI-32982 following a single IV and PO dose in mice

Parameter	IV (5 mg/kg)	PO (10 mg/kg)
$t_{1/2}$ (h)	0.5 ± 0.2	0.3
t_{max} (h)	NA	0.4 ± 0.1
Cmax (ng/mL)	NA	38.9 ± 18.7
AUC_{last} (ng·h/mL)	1282.7 ± 528.2	19.2 ± 7.4
AUC_{inf} (ng·h/mL)	1287.2 ± 526.8	15.4
MRT (h)	0.2 ± 0.1	0.6
V_z (L/kg)	3.5 ± 2.9	NA
CL (L/h/kg)	4.4 ± 1.8	NA
V_{ss} (L/kg)	1.2 ± 0.9	NA
F (%)	NA	0.7 ± 0.3

PK parameters were calculated by non-compartment analysis using PKSolver. PK parameters requiring terminal elimination rate constant (k), such as $t_{1/2}$, AUC_{inf} and MRT(h) could not be determined due to lack of terminal phase in the plasma concentration-time curves. NA: not applicable. ND: not determined.

Analysis of BC-DXI-32982 in Tumor Tissues excised from mice

Analytical method

1) Pretreatment of tissue: Tumor tissues were homogenized in buffer and diluted with acetonitrile following vortexing for 10 min and centrifugation (15,000 rpm, 4 °C) for 10 min. The supernatant was transferred to LC vial for analysis.

2) LC-MS/MS

- Instrument: Mass sepctrometry (Agilent 6460) with HPLC (Agilent 1200)
- Elution condition: Column (UK-C18, 100 X 2 mm, 3 μm, UNISON, 35 °C); Mobile Phase A (10 mM ammonium formate in water), Mobile Phase B (100% acetonitrile)
- Flow rate: 0.35 mL/min, Total run time: 5.5 min, Injection volume: 5 μL

Time (min)	%A	%B
0.00	80	20
0.30	80	20
2.30	0	100
2.50	0	100
2.60	80	20
5.50	80	20

3) Source/ Gas parameters: ion source (ESI); GasTemp (350 °C); Gas flow (10 L/min); Nebulizer (45 psi)

4) Internal standard: disopyramide

5) Detector: EMV 1850

Result

Concentration of BC-DXI-32982 (ng/g)			
HCC1588	A549	Male	Female
23571 ± 44421	8044 ± 4524	11242 ± 4654	7917 ± 3165

Panel Screen on BC-DXI-32982

Analytical method

SafetyScreen44-Panlabs serviced by eurofins was performed.

1) Cholinesterase, Acetyl, ACES (104010)

- Source: Human recombinant HEK-293 cells
- Substrate: 700 μM Acetylthiocholine
- Vehicle: 1.0% DMSO
- Significance Criteria: ≥50% of max stimulation or inhibition
- Pre-Incub. Time/Temp: 15 minutes @ 25°C
- Incubation Time/Temp: 20 minutes @ 25°C
- Incubation Buffer: 0.1 M Sodium Phosphate, pH 7.4
- Quantitation Method: Spectrophotometric quantitation of Thiocholine

2) Adrenergic β_2 (204110)

- Source: Human recombinant CHO cells
- Vehicle: 1.0% DMSO
- Incubation Time/Temp: 60 minutes @ 25°C
- Incubation Buffer: 50 mM Tris-HCl, pH 7.4, 0.5 mM EDTA, 5.0 mM $MgCl_2$, 120 mM NaCl

- Kd: 0.44 nM
- Ligand: 0.2 nM [^{3}H] CGP-12177
- Non-Specific Ligand: 10.0 μM ICI-118551
- Specific Binding: 95%
- Quantitation Method: Radioligand Binding
- Significance Criteria: ≥50% of max stimulation or inhibition
- Bmax: 0.44 pmole/mg Protein

3) Calcium Channel L-Type, Dihydropyridine (214600)
- Source: Wistar Rat cerebral cortex
- Vehicle: 1.0% DMSO
- Incubation Time/Temp: 90 minutes @ 25°C
- Incubation Buffer: 50 mM Tris-HCl, pH 7.4
- Kd: 0.18 nM
- Ligand: 0.1 nM [^{3}H] Nitrendipine
- Non-Specific Ligand: 1.0 μM Nitrendipine
- Specific Binding: 91%
- Quantitation Method: Radioligand Binding
- Significance Criteria: ≥50% of max stimulation or inhibition
- Bmax: 0.23 pmole/mg Protein

4) Cholecystokinin CCK_1 (CCK_A) (218030)
- Source: Human recombinant 1321-N1 cells
- Vehicle: 1.0% DMSO
- Incubation Time/Temp: 3 hours @ 25°C

- Incubation Buffer: 50 mM HEPES, pH 7.4, 5 mM $MgCl_2$, 1 mM $CaCl_2$, 0.1% BSA
- Kd: 0.59 nM
- Ligand: 0.11 nM [^{125}I] CCK-8
- Non-Specific Ligand: 1.0 μM L-364,718
- Specific Binding: 85%
- Quantitation Method: Radioligand Binding
- Significance Criteria: ≥50% of max stimulation or inhibition
- Bmax: 2.30 pmole/mg Protein

5) Sodium Channel, Site 2 (279510)

- Source: Wistar Rat brain (minus cerebellum)
- Vehicle: 1.0% DMSO
- Incubation Time/Temp: 60 minutes @ 37°C
- Incubation Buffer: 50 mM HEPES, 50 mM Tris-HCl, pH 7.4, 130 mM Choline Chloride, 5.4 mM KCl, 0.8 mM $MgCl_2$, 5.5 mM Glucose, 40 μg/mL LqTX
- Kd: 0.052 μM
- Ligand: 5.0 nM [^{3}H] Batrachotoxinin
- Non-Specific Ligand: 100 μM Veratridine
- Specific Binding: 77%
- Quantitation Method: Radioligand Binding
- Significance Criteria: ≥50% of max stimulation or inhibition
- Bmax: 0.7 pmole/mg Protein

Result

Among 44 assays using 10 μM of BC-32982, significant responses above 50% inhibition were noted in 5 primary assays listed below.

Cat #	Assay names	Species	Conc.	% Inh.
104010	Cholinesterase, Acetyl, ACES	Hum	10 μM	66
204110	Adrenergic β_2	Hum	10 μM	55
214600	Calcium Channel L-Type, Dihydropyridine	Rat	10 μM	63
218030	Cholecystokinin CCK_1 (CCK_A)	Hum	10 μM	54
279510	Sodium Channel, Site 2	Rat	10 μM	75

In vivo toxicity by 14 day repeated treatment of BC-DXI-32982

Analytical method

1) Animal: ICR male mice

2) Administration: Tale intravenous (IV) injection once a day for 14 days

3) Dosage: Vehicle, 10 mg/kg, 50 mg/kg (5 mice per each treated group)

4) Dosing solution: 1 mg/mL in dimethylacetamide/Tween 80/water (10/10/80 vol%)

5) Observation

- Clinical pathology: Blood and biochemical analysis
- Organ and Tissue: Lung, brain, liver, kidney, stomach, spleen and thymus

Results

1) Mean body weight over time: No significant difference between vehicle and chemical treated groups

2) Mortality: No death

3) Clinical signs: No abnormalities detected

4) Hematology

- In 50 mg/kg treated group, there was a tendency that AST and ALT was increased without significance

- In 50 mg/kg treated group, LUC and monocyte were significantly increased

5) Macroscopic Findings: No significant abnormalities detected

6) Organ weight: There was a tendency that liver weight with gallbladder seemed to be increased without significance.

7) Microscopic Findings

- Liver: Hypertropy of hepatocyte was observed in 10 and 50 mg/kg treated group

- Lung: Infiltration of missed cell was observed in 50 mg/kg treated group

Supplementary Note 2

Experimental Procedures

Chemistry. General.

All the commercial chemicals were of reagent grade and were used without further purification. Solvents were dried with standard procedures. All the reactions were carried out under an atmosphere of dried argon in flame-dried glassware. The proton nuclear magnetic resonance (^{1}H-NMR) spectra were determined on a Varian (400 MHz) spectrometer (Varian Medical Systems, Inc., Palo Alto, CA, USA). ^{13}C-NMR spectra were recorded on a Varian (100 MHz) spectrometer. Multiplicities of NMR signals were reported using different abbreviations like singlet (s), doublet (d), triplet (t), quartet (q) and multiplet (m). The chemical shifts are provided in parts per million (ppm) downfield with coupling constants in hertz (Hz). ^{19}F NMR spectra were recorded on a Varian (376 MHz) spectrometer. The mass spectra were recorded using high-resolution mass spectrometry (HRMS) (electron ionization MS) obtained on a JMS-700 mass spectrometer (Jeol, Japan) or using HRMS (electrospray ionization MS) obtained on a G2 QTOF mass spectrometer. Melting points were measured on a Fisherbrand digital melting point apparatus. The products from all the reactions were purified by flash column chromatography using silica gel 60 (230–400 mesh Kieselgel 60). Additionally, thin-layer chromatography on 0.25-mm silica plates (E. Merck; silica gel 60 F254) was used to monitor reactions. Final product purity was checked by reversed phase high-pressure liquid chromatography (RP-HPLC), performed on a Waters Corp. HPLC system equipped with an ultraviolet (UV) detector set at 254 nm. The mobile phases used were: (A) H_2O containing 0.05% trifluoroacetic acid; and (B) CH_3CN. HPLC employed a YMC Hydrosphere C18 (HS-

302) column (5-μm particle size, 12-nm pore size) that was 4.6 mm in diameter × 150 mm in size with a flow rate of 1.0 mL/min. Compound purity was assessed using the following method: CH_3CN:0.05% TFA in H_2O from 25:75 to 100:0. The purity of all biologically evaluated compounds was >99%.

5-(4-Methoxy-3-methylphenyl)thiophene-2-carbaldehyde (3)

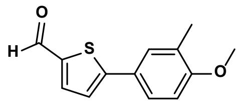

Pd(dppf)Cl_2.CH_2Cl_2 (0.42 g, 0.52 mmol) was added to a degassed solution of 5-bromothiophene-2-carboxaldehyde (**1**) (2.0 g, 10.4 mmol), 4-methoxy-3-methylphenylboronic acid (**2**) (2.08 g, 12.5 mmol), and K_3PO_4 (4.44 g, 20.9 mmol) in DMF:H_2O (4:1) (20 mL). The reaction mixture was heated at 100 ºC for 2 h. The reaction mass was cooled to rt, diluted with H_2O and extracted with EtOAc. The combined organic layer was washed with H_2O, brine, dried over $MgSO_4$, filtered and concentrated in vacuo. Purification by flash column chromatography (silica gel, 0 to 5% EtOAc in hexanes) gave **3** as a yellow solid (2.20 g, 90%). ^{1}H NMR (400 MHz, $CDCl_3$) δ 9.85 (s, 1H), 7.70 (d, *J* = 4.0 Hz, 1H), 7.49 (d, *J* = 8.4 Hz, 1H), 7.45 (s, 1H), 7.29 (d, *J* = 4.0 Hz, 1H), 6.86 (d, *J* = 8.8 Hz, 1H), 3.87 (s, 3H), 2.26 (s, 3H); HRMS (ESI) (*m/z*): $[M + H]^+$ calcd (Calculated). for $C_{13}H_{13}O_2S$, 233.0636; found, 233.0626.

2-Fluoro-*N*-((5-(4-methoxy-3-methylphenyl)thiophen-2-yl)methyl)aniline (4)

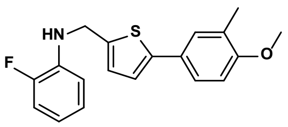

To a solution of aldehyde (**3**) (1.62 g, 6.97 mmol) and 2-fluoroaniline (0.56 mL, 5.79 mmol) in CH_2Cl_2, catalytic amount of acetic acid was added and reaction mixture

was stirred at rt till the complete formation of imine which was monitored by TLC (Thin-layer chromatography). After evaporating CH_2Cl_2 under vacuum, the obtained residue was dissolved in MeOH, $NaCNBH_3$ (1.75 g, 27.9 mmol) was added and reaction mixture was stirred at rt for 12 h. After evaporating MeOH under vacuum, the reaction mixture was diluted with H_2O and extracted with EtOAc. The combined organic layers were washed with H_2O and brine, dried over $MgSO_4$, and evaporated under vacuum to afford a crude product. Purification by flash column chromatography (silica gel, 0−5% EtOAc in hexanes) afforded **4** as a white solid (1.52 g, 67%). ^1H NMR (400 MHz, $CDCl_3$) δ 7.35 (d, *J* = 8.8 Hz, 1H), 7.34 (s, 1H), 7.03−6.96 (m, 3H), 6.94 (d, *J* = 3.6 Hz, 1H), 6.80 (d, *J* = 8.0 Hz, 2H), 6.66 (q, *J* = 6.8 Hz, 1H), 4.53 (d, *J* = 5.6 Hz, 2H), 4.34 (br, 1H), 3.84 (s, 3H), 2.23 (s, 3H); HRMS (ESI) (*m/z*): $[M + H]^+$ calcd. for $C_{19}H_{19}FNOS$, 328.1171; found 328.1182.

***N*-(2-Fluorophenyl)-*N*-((5-(4-methoxy-3-methylphenyl)thiophen-2-yl)methyl)quinoxaline-2-carboxamide (5a) (BC-DXI-32982).**

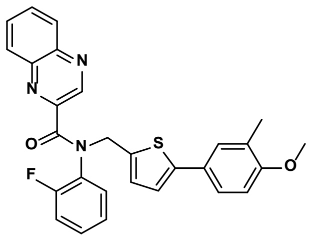

2-Quinoxaloyl chloride (0.35 g, 1.83 mmol) was added to a solution of compound **4** (0.30 g, 0.91 mmol) and triethylamine (0.26 mL, 1.83 mmol) in CH_2Cl_2 at 0 °C under argon atmosphere. Reaction mixture was warmed to rt and stirred for 12 h. The reaction mixture was diluted with CH_2Cl_2, washed with sat (saturated). $NaHCO_3$, H_2O, brine, dried over $MgSO_4$ and concentrated in vacuo. Purification by flash column chromatography (silica gel, 0−25% EtOAc in hexanes) yielded **5a** as a dark yellow

solid (0.42 g, 94%). mp: 102−104 °C. ^{1}H NMR (400 MHz, $CDCl_3$) δ 9.23 (s, 1H), 8.01 (d, *J* = 8.0 Hz, 1H), 7.72−7.60 (m, 3H), 7.36 (d, *J* = 7.2 Hz, 1H), 7.35 (s, 1H), 7.16−7.10 (m, 1H), 7.07 (td, *J* = 7.8, 1.4 Hz, 1H), 6.97 (d, *J* = 3.6 Hz, 1H), 6.95−6.90 (m, 2H), 6.84 (d, *J* = 3.6 Hz, 1H), 6.78 (d, *J* = 8.8 Hz, 1H), 5.57 (d, *J* = 14.8 Hz, 1H), 4.95 (d, *J* = 14.8 Hz, 1H), 3.81 (s, 3H), 2.22 (s, 3H); ^{13}C NMR (100 MHz, $CDCl_3$) δ 166.3, 158.9, 157.4, 156.4, 147.1, 145.4, 144.7, 142.1, 140.1, 136.2, 131.2, 130.4, 130.2, 129.9, 129.8, 129.7, 129.5, 129.4, 129.0, 128.9, 128.2, 126.9, 126.5, 124.4, 124.3, 124.2, 121.0, 116.2, 116.0, 110.1, 55.4, 48.3, 16.2; ^{19}F NMR (376 MHz, $CDCl_3$) δ -(121.12−121.18) (m); HRMS (ESI) (*m/z*): $[M + H]^+$ calcd. for $C_{28}H_{23}FN_3O_2S$, 484.1495; found, 484.1485; RP-HPLC purity 99.2% at 254 nm, R_t = 24.6 min.

2-Chloro-*N*-(2-fluorophenyl)-*N*-((5-(4-methoxy-3-methylphenyl)thiophen-2-yl)methyl)acetamide (5b)

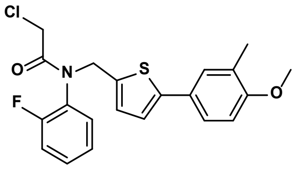

Chloroacetyl chloride (0.04 mL g, 0.61 mmol) was slowly added to a solution of compound **4** (0.10 g, 0.30 mmol) and triethylamine (0.12 mL, 0.91 mmol) in CH_2Cl_2 at 0 °C under argon atmosphere. Reaction mixture was warmed to rt and stirred for 12 h. The reaction mixture was diluted with CH_2Cl_2, washed with sat. $NaHCO_3$, H_2O, brine, dried over $MgSO_4$ and evaporated under vacuum to afford a crude product. Purification by flash column chromatography (silica gel, 0−20% EtOAc in hexanes) afforded **5b** as a colorless oil (75.0 mg, 61%). ^{1}H NMR (400 MHz, $CDCl_3$) δ 7.41−7.33 (m, 3H), 7.21−7.07 (m, 3H), 6.95 (d, *J* = 3.6 Hz, 1H), 6.80 (d, *J* = 7.6 Hz, 1H), 6.72 (d, *J* = 3.6 Hz, 1H), 5.41 (d, *J* = 15.2 Hz, 1H), 4.56 (d, *J* = 14.8 Hz, 1H),

3.87 (d, J = 5.2 Hz, 2H), 3.84 (s, 3H), 2.23 (s, 3H); HRMS (ESI) (m/z): $[M + H]^+$ calcd. for $C_{21}H_{20}ClFNO_2S$, 404.0887; found, 404.0881.

2-((2-Fluorophenyl)((5-(4-methoxy-3-methylphenyl)thiophen-2-yl)methyl)amino)-2-oxoethyl 5-((3a*S*,4*S*,6a*R*)-2-oxohexahydro-1*H*-thieno[3,4-*d*]imidazol-4-yl)pentanoate (6) (Biotin-32982 #1)

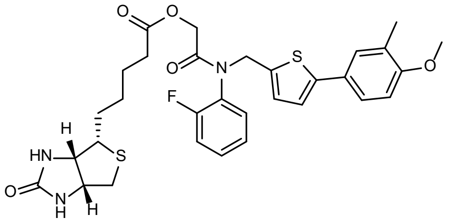

Compound **5b** (70.0 mg, 0.17 mmol) was added to a suspension of D-biotin (51.0 mg, 0.20 mmol), cat. KI, and K_2CO_3 (72.0 mg, 0.52 mmol) in DMF and the reaction mixture was stirred at rt for 12 h under argon atmosphere. The reaction mixture was quenched with H_2O and extracted with EtOAc. The combined organic layers were washed with brine, dried over $MgSO_4$ and concentrated. Purification by flash column chromatography (silica gel, 0−3% MeOH in CH_2Cl_2) afforded **6** as a white solid (40.0 mg, 38%). mp: 73−75 °C. ^1H NMR (400 MHz, $CDCl_3$) δ 7.41−7.32 (m, 3H), 7.22−7.12 (m, 3H), 6.94 (d, J = 3.6 Hz, 1H), 6.80 (d, J = 8.4 Hz, 1H), 6.73 (t, J = 2.8 Hz, 1H), 5.97 (d, J = 24.4 Hz, 1H), 5.37 (dd, J = 33.0, 15.0 Hz, 1H), 5.01 (s, 1H), 4.60 (dd, J = 33.2, 15.2 Hz, 1H), 4.54−4.48 (m, 1H), 4.41−4.25 (m, 3H), 3.84 (s, 3H), 3.15 (q, J = 6.4 Hz, 1H), 2.96−2.88 (m, 1H), 2.76 (d, J = 12.8 Hz, 1H), 2.53−2.36 (m, 2H), 2.22 (s, 3H), 1.79−1.70 (m, 4H), 1.56−1.45 (m, 2H); ^{13}C NMR (100 MHz, $CDCl_3$) δ 173.2, 166.7, 163.7, 159.4, 157.5, 156.9, 145.2, 136.38, 136.33, 130.9, 130.8, 130.7, 128.65, 128.60, 128.2, 127.1, 127.0, 126.9, 126.6, 125.25, 125.22, 124.2, 121.1, 117.2, 116.9, 110.2, 61.8, 61.2, 60.2, 55.7, 55.4, 47.4, 40.5, 33.4, 28.2, 28.1, 24.8, 16.2; ^{19}F NMR (376 MHz, $CDCl_3$) δ -(120.24−120.27) (m); HRMS (ESI) (m/z):

[M + H]$^+$ calcd. for $C_{31}H_{35}FN_3O_5S_2$, 612.2002; found, 612.2012; RP-HPLC purity >99.9% at 254 nm, R_t = 18.8 min.

***N*-(2-Fluorophenyl)-*N*-((5-(4-methoxy-3-methylphenyl)thiophen-2-yl)methyl)-4-nitrobenzamide (7)**

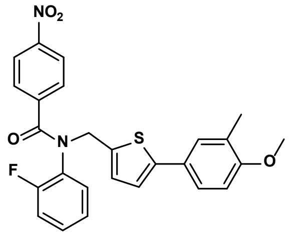

4-Nitrobenzoyl chloride (0.59 g, 3.20 mmol) was added to a solution of compound **4** (0.35 g, 1.06 mmol) and triethylamine (0.45 mL, 3.20 mmol) in CH_2Cl_2 at 0 °C under argon atmosphere. Reaction mixture was warmed to rt and stirred for 12 h. The reaction mixture was diluted with CH_2Cl_2, washed with sat. $NaHCO_3$, H_2O, brine, dried over $MgSO_4$ and evaporated under vacuum to afford a crude product. Purification by flash column chromatography (silica gel, 0−20% EtOAc in hexanes) afforded **7** as yellow solid (0.46 g, 90%). ^{1}H NMR (400 MHz, $CDCl_3$) δ 8.02 (d, *J* = 8.0 Hz, 2H), 7.51 (d, *J* = 8.8 Hz, 2H), 7.36 (d, *J* = 8.4 Hz, 1H), 7.35 (s, 1H), 7.21−7.16 (m, 1H), 6.99−6.95 (m, 4H), 6.80 (d, *J* = 8.0 Hz, 1H), 6.77 (d, *J* = 3.2 Hz, 1H), 5.50 (d, *J* = 14.8 Hz, 1H), 4.82 (d, *J* = 14.2 Hz, 1H), 3.83 (s, 3H), 2.23 (s, 3H); HRMS (ESI) (*m/z*): [M - H]$^-$ calcd. for $C_{26}H_{20}FN_2O_4S$, 475.1128; found, 475.1147.

4-Amino-*N*-(2-fluorophenyl)-*N*-((5-(4-methoxy-3-methylphenyl)thiophen-2-yl)methyl)benzamide (8)

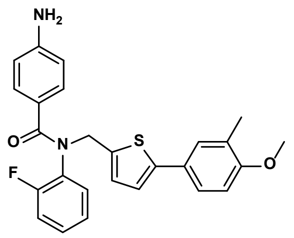

To a degassed solution of **7** (0.20 g, 0.42 mmol) in EtOAc, 10% Pd/C (10% w/w) (44.0 mg, 10 mol%) was slowly added and the reaction was stirred under hydrogen atmosphere overnight. After completion of the reaction, the reaction mass was filtered through a celite pad, concentrated and purified by flash column chromatography (silica gel, 0−30% EtOAc in hexanes) gave **8** as off white solid (0.13 g, 68%). ^{1}H NMR (400 MHz, $CDCl_3$) δ 7.33 (d, *J* = 7.2 Hz, 2H), 7.25 (s, 1H), 7.20 (d, *J* = 8.8 Hz, 1H), 7.16−7.10 (m, 1H), 6.98 (q, *J* = 8.8 Hz, 1H), 6.97−6.89 (m, 3H), 6.78 (d, *J* = 8.8 Hz, 1H), 6.73 (t, *J* = 3.2 Hz, 1H), 6.65 (d, *J* = 8.4 Hz, 1H), 6.38 (d, *J* = 8.4 Hz, 1H), 5.44 (br, 1H), 4.77 (br, 1H), 3.82 (s, 3H), 2.22 (s, 3H) (-NH_2 protons were not observed); HRMS (ESI) (*m/z*): $[M + H]^+$ calcd. for $C_{26}H_{24}FN_2O_2S$, 447.1542; found, 447.1542.

4-(2-Chloroacetamido)-*N*-(2-fluorophenyl)-*N*-((5-(4-methoxy-3-methylphenyl)thiophen-2-yl)methyl)benzamide (9)

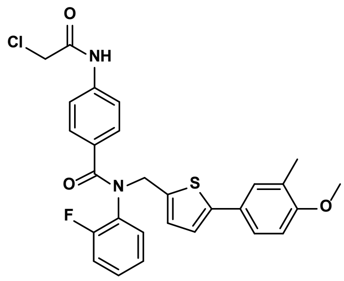

Chloroacetyl chloride (0.05 mL, 0.65 mmol) was slowly added to a solution of compound **8** (0.14 g, 0.32 mmol) and triethylamine (0.14 mL, 0.97 mmol) in CH_2Cl_2 at 0 °C under argon atmosphere. Reaction mixture was stirred for 12 h slowly rising

to rt. The reaction mixture was diluted with CH_2Cl_2, washed with sat. $NaHCO_3$, H_2O, brine, dried over $MgSO_4$ and evaporated under vacuum to afford a crude product. Purification by flash column chromatography (silica gel, 0−25% EtOAc in hexanes) afforded **9** as a light brown solid (88.0 mg, 51%). ^{1}H NMR (400 MHz, $CDCl_3$) δ 8.19 (s, 1H), 7.39 (s, 4H), 7.35 (s, 2H), 7.19−7.13 (m, 1H), 6.99 (t, *J* = 9.0 Hz, 1H), 6.96−6.92 (m, 3H), 6.80 (d, *J* = 8.0 Hz, 1H), 6.75 (d, *J* = 3.6 Hz, 1H), 5.50 (d, *J* = 14.4 Hz, 1H), 4.79 (d, *J* = 15.6 Hz, 1H), 4.13 (s, 2H), 3.84 (s, 3H), 2.23 (s, 3H); HRMS (ESI) (*m/z*): $[M + H]^+$ calcd. for $C_{28}H_{25}ClFN_2O_3S$, 523.1258; found, 523.1251.

2-((4-((2-Fluorophenyl)((5-(4-methoxy-3-methylphenyl)thiophen-2-yl)methyl)carbamoyl)phenyl)amino)-2-oxoethyl 5-((3a*S*,4*S*,6a*R*)-2-oxohexahydro-1*H*-thieno[3,4-*d*]imidazol-4-yl)pentanoate (10) (Biotin-32982 #2)

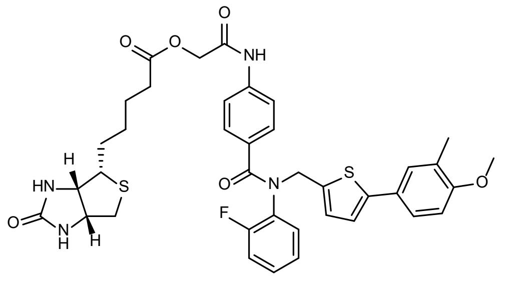

Compound **9** (40.0 mg, 0.08 mmol) was added to a suspension of D-biotin (19.0 mg, 0.08 mmol), cat. KI, and K_2CO_3 (32.0 mg, 0.23 mmol) in DMF and the reaction mixture was stirred at rt for 12 h under argon atmosphere. The reaction mixture was quenched with H_2O and extracted with EtOAc. The combined organic layers were washed with brine, dried over $MgSO_4$ and concentrated. Purification by flash column chromatography (silica gel, 0−5% MeOH in CH_2Cl_2) yielded **10** as a white solid (40.0 mg, 36%). mp: 108−110 °C. ^{1}H NMR (400 MHz, $CDCl_3$) δ 8.92 (s, 1H), 7.43 (d, *J* = 8.0 Hz, 2H), 7.36−7.31 (m, 4H), 7.17−7.11 (m, 1H), 6.99−6.92 (m, 4H), 6.79 (d, *J* = 8.4 Hz, 1H), 6.73 (d, *J* = 3.6 Hz, 1H), 6.41 (s, 1H), 5.50 (s, 1H), 5.44 (d, *J* = 14.8 Hz,

1H), 4.79 (d, *J* = 14.8 Hz, 1H), 4.56 (q, *J* = 11.5 Hz, 2H), 4.41 (q, *J* = 4.1 Hz, 1H), 4.22 (q, *J* = 4.1 Hz, 1H), 3.83 (s, 3H), 3.07 (q, *J* = 6.4 Hz, 1H), 2.81 (dd, *J* = 12.8, 4.8 Hz, 1H), 2.64 (d, *J* = 12.8 Hz, 1H), 2.44−2.35 (m, 2H), 2.22 (s, 3H), 1.69−1.57 (m, 4H), 1.44−1.36 (m, 2H); ^{13}C NMR (100 MHz, $CDCl_3$) δ 172.8, 170.3, 165.8, 163.8, 158.6, 157.4, 156.1, 145.0, 139.2, 136.9, 130.9, 130.3, 129.3, 129.2, 128.5, 128.4, 128.1, 126.9, 126.6, 124.64, 124.61, 124.1, 120.9, 118.8, 116.6, 116.4, 110.0, 63.2, 61.7, 60.1, 55.6, 55.4, 48.2, 40.5, 33.2, 27.92, 27.89, 24.6, 16.2; ^{19}F NMR (376 MHz, $CDCl_3$) δ -120.32 (br s, Broad singlet); HRMS (ESI) (*m/z*): $[M + H]^+$ calcd. for $C_{38}H_{40}FN_4O_6S_2$, 731.2373; found, 731.2374; RP-HPLC purity 99.1% at 254 nm, R_t = 18.9 min.
