# Supplementary material for: AIMP2-DX2 provides therapeutic interface to control KRAS-driven tumorigenesis
Source: Nat Commun. 2022 May 11;13:2572. doi: 10.1038/s41467-022-30149-2 (PMC9095880; doi:10.1038/s41467-022-30149-2)
Supplement: Supplementary file 3 — Description of Additional Supplementary Files [file 41467_2022_30149_MOESM3_ESM.pdf]

Description of Additional Supplementary Files

File Name: Supplementary Movie 1
Description: **MD trajectory for the DX2-KRAS binding event during the last 100 ns.** Protein structures of the DX2 GST domain (light green) and KRAS4B (magenta) are shown as ribbon models with binding residues as stick models. The trajectory frames were smoothed with a window size of 5 frames.

File Name: Supplementary Movie 2
Description: **MD trajectory for the DX2-BC-DXI-32982 binding event.** DXI is represented as a stick model and key interacting residues (Y47, G48, V54, I119, and K129) are displayed as a thin stick model. Visualization of protein movement was difficult due to thermal fluctuation. Hence, the movie was recorded with trajectory smoothed option by averaging 5 frames of total ~2500 frames for 250 ns.
