# Supplementary material for: AIMP2-DX2 provides therapeutic interface to control KRAS-driven tumorigenesis
Source: Nat Commun. 2022 May 11;13:2572. doi: 10.1038/s41467-022-30149-2 (PMC9095880; doi:10.1038/s41467-022-30149-2)
Supplement: Supplementary file 6 — Reporting Summary [file 41467_2022_30149_MOESM6_ESM.pdf]

nature portfolio

Corresponding author(s): Kyeong Lee & Sunghoon Kim

Last updated by author(s): Mar 16, 2022

Reporting Summary

Nature Portfolio wishes to improve the reproducibility of the work that we publish. This form provides structure for consistency and transparency in reporting. For further information on Nature Portfolio policies, see our Editorial Policies and the Editorial Policy Checklist.

Statistics

For all statistical analyses, confirm that the following items are present in the figure legend, table legend, main text, or Methods section.

n/a	Confirmed	
☐	☒	The exact sample size (*n*) for each experimental group/condition, given as a discrete number and unit of measurement
☐	☒	A statement on whether measurements were taken from distinct samples or whether the same sample was measured repeatedly
☐	☒	The statistical test(s) used AND whether they are one- or two-sided<br>*Only common tests should be described solely by name; describe more complex techniques in the Methods section.*
☒	☐	A description of all covariates tested
☒	☐	A description of any assumptions or corrections, such as tests of normality and adjustment for multiple comparisons
☐	☒	A full description of the statistical parameters including central tendency (e.g. means) or other basic estimates (e.g. regression coefficient) AND variation (e.g. standard deviation) or associated estimates of uncertainty (e.g. confidence intervals)
☐	☒	For null hypothesis testing, the test statistic (e.g. *F*, *t*, *r*) with confidence intervals, effect sizes, degrees of freedom and *P* value noted<br>*Give P values as exact values whenever suitable.*
☒	☐	For Bayesian analysis, information on the choice of priors and Markov chain Monte Carlo settings
☒	☐	For hierarchical and complex designs, identification of the appropriate level for tests and full reporting of outcomes
☒	☐	Estimates of effect sizes (e.g. Cohen's *d*, Pearson's *r*), indicating how they were calculated

Our web collection on statistics for biologists contains articles on many of the points above.

Software and code

Policy information about availability of computer code

Data collection	Discovery Studio (DS) 2018 software, CHARMM-GUI program (version 2.0), CHARMM General Force Field (CGenFF) program (version 1.0.0) were used for molecular dynamics (MD) simulation system preparation. GROMACS software (version 2016) with charmm36 force field, and the Plumed plugin version 2.4 were used to acquire MD simulation trajectory. Nosé-Hoover thermostat, Parrinello-Rahman barostat, Particle Mesh Ewald (PME) method, LINCS algorithm are implemented in the GROMACS software.
Data analysis	ZDOCK protein-protein docking simulation of Discovery Studio (DS) 2018 software was used to generate initial structure. Discovery Studio (DS) 2018 software was used for graphical representation. For recording movie of MD trajectory, Visual Molecular Dynamics (VMD) program (version 1.9.4a12) was used. GROMACS software (version 2016) was used to measure root-mean-square deviation (RMSD), distance, energy.

For manuscripts utilizing custom algorithms or software that are central to the research but not yet described in published literature, software must be made available to editors and reviewers. We strongly encourage code deposition in a community repository (e.g. GitHub). See the Nature Portfolio guidelines for submitting code & software for further information.

Data

Policy information about availability of data

All manuscripts must include a data availability statement. This statement should provide the following information, where applicable:

- Accession codes, unique identifiers, or web links for publicly available datasets
- A description of any restrictions on data availability
- For clinical datasets or third party data, please ensure that the statement adheres to our policy

All data are available within this article and the Supplementary Information files. The mass spectrometry proteomics data generated in this study have been deposited in the ProteomeXchange Consortium via the PRIDE partner repository under accession code PXD029839. Source data are provided with this paper.

Field-specific reporting

Please select the one below that is the best fit for your research. If you are not sure, read the appropriate sections before making your selection.

☒ Life sciences ☐ Behavioural & social sciences ☐ Ecological, evolutionary & environmental sciences

For a reference copy of the document with all sections, see nature.com/documents/nr-reporting-summary-flat.pdf

Life sciences study design

All studies must disclose on these points even when the disclosure is negative.

Sample size	For animal experiments, the rational for analyzing multiple cells/fields from 4-6 biologically independent mice in each group (exact numbers mentioned in figure legends) was based on a resource efficient strategy common across relevant publications (Karkampouna et al. 2021 Nat Commun, PMID: 33602919; Harney et al. 2015 Cancer Disc, PMID: 26269515; Karagiannis et al. 2017 Sci Transl Med, PMID: 28679654). For all other experiments, cells/fields measurement were made in at least 3 biologically independent replicates and is also common across relevant publications (Adhikari et al. 2021 Nat Commun, PMID: 34504076; Karkampouna et al. 2021 Nat Commun, PMID: 33602919; Lito et al. 2016 Science, PMID: 26841430).
Data exclusions	No data were excluded.
Replication	All experiments were repeated independently with similar results for at least three times.
Randomization	All the samples except for immunoblotting were randomized. Independent experiments like immunoblotting cannot be randomized because number of sample for immunoblotting per experiment was only one, but the experiments were independently repeated three times with similar results.
Blinding	All the sample preparation except for immunoblotting were blinded. Independent experiments like immunoblotting cannot be blinded because number of sample for immunoblotting per experiment was only one, but the experiments were independently repeated three times with similar results.

Reporting for specific materials, systems and methods

We require information from authors about some types of materials, experimental systems and methods used in many studies. Here, indicate whether each material, system or method listed is relevant to your study. If you are not sure if a list item applies to your research, read the appropriate section before selecting a response.

Materials & experimental systems

n/a	Involved in the study
☐	☒ Antibodies
☐	☒ Eukaryotic cell lines
☒	☐ Palaeontology and archaeology
☐	☒ Animals and other organisms
☐	☒ Human research participants
☒	☐ Clinical data
☒	☐ Dual use research of concern

Methods

n/a	Involved in the study
☒	☐ ChIP-seq
☒	☐ Flow cytometry
☒	☐ MRI-based neuroimaging

Antibodies

Antibodies used	Anti-DX2 (Mouse), 1:1000, Curebio, NMS-02-0012, 115<br>Anti-AIMP2 (Mouse), 1:1000, Curebio, NMS-02-0011<br>Anti-FLAG (Mouse), 1:5000, Sigma, F3165, clone M2<br>Anti-Actin (Mouse), 1:5000, Sigma, A1978, clone AC-15<br>Anti-pan-RAS (Mouse), 1:1000, Sigma, MABS195, clone RAS 10<br>Anti-Strep (murine monoclonal antibody for detection of Strep-tag and coupled to horseradish peroxidase (HRP)), 1:10000, IBA, 2-1509-001<br>Anti-RCE1 (Rabbit), 1:500, Novus Biologicals, NBP1-59922<br>Anti-KRAS (Rabbit), 1:50, Novus Biologicals, NBP2-33579<br>Anti-p14ARF (Mouse), 1:500, Merck, MAB3782, clone 4C6/4<br>Anti-HRAS (Rabbit), 1:500, Thermo Fisher Scientific, 18295-1-AP<br>Anti-PRDX1 (Rabbit), 1:1000, Thermo Fisher Scientific, PA3-750<br>Anti-p-ERK (Rabbit), 1:1000, Cell Signaling Technology, #9101<br>Anti-ERK (Rabbit), 1:1000, Cell Signaling Technology, #4695, 137F5<br>Anti-p-Akt (Rabbit), 1:1000, Cell Signaling Technology, #4060, D9E<br>Anti-Akt (Rabbit), 1:1000, Cell Signaling Technology, #9272

	Anti-EGFR (Rabbit), 1:1000, Cell Signaling Technology, #4267, D38B1<br>Anti-p-EGFR (Rabbit), 1:1000, Cell Signaling Technology, #3777, D7A5<br>Anti-cleaved caspase-3 (Rabbit), 1:1000, Cell Signaling Technology, #9661<br>Anti-KRAS (Mouse), 1:500, Santa Cruz Biotechnology, sc-30, F234<br>Anti-GFP (Mouse), 1:1000, Santa Cruz Biotechnology, sc-9996, B-2<br>Anti-HSP90 (Mouse), 1:1000, Santa Cruz Biotechnology, sc-13119, F-8<br>Anti-pan-cadherin (Mouse), 1:500, Santa Cruz Biotechnology, sc-515872, E-11<br>Anti-Smurf2 (Mouse), 1:500, Santa Cruz Biotechnology, sc-518164, C-5<br>Anti-ubiquitin (Mouse), 1:500, Santa Cruz Biotechnology, sc-53509, P4G7<br>Anti-HA (Mouse), 1:1000, Santa Cruz Biotechnology, sc-7392, F-7<br>Anti-myc (Mouse), 1:1000, Santa Cruz Biotechnology, sc-40, 9E10<br>Anti-HSP70 (Mouse), 1:1000, Santa Cruz Biotechnology, sc-24, W27<br>Anti-YY1 (Mouse), 1:1000, Santa Cruz Biotechnology, sc-7341, H-10<br>Anti-E-cadherin (Mouse), 1:500, Santa Cruz Biotechnology, sc-8426, G-10
Validation	All the antibodies used in this work have been validated by the companies where these antibodies were purchased from, by western blot using human and mouse cell lysates samples.

Eukaryotic cell lines

Policy information about cell lines

Cell line source(s)	COLO-205, HCT-8, KM-12, SNU-C4, SW-403, NCI-H747, SNU-407, NCI-H1666, NCI-H1650, Calu-6, NCI-H441, HCC-1588, and SNU-410 cell lines were purchased from the Korean Cell Line Bank.<br>CCD18CO, HCT-116, DLD-1, LoVo, WI-26, H460, AsPC-1, Panc10.05, BxPC3, SU.86.86, CFPAC-1, MIA-PaCa2, H69, WI-38, HCC44, H1975, HCC2108, H1299, H1792, H226, A549, HCC827, H520, CaCo2, Panc1, 293T, and CHO-K1 cell lines were obtained from the BioBank of Medicinal Bioconvergence Research Center (Biocon).<br>Lysates of HeLa cells in which the KRAS gene was knocked out using CRISPR/Cas9 were purchased from Abcam
Authentication	Short tandem repeat profiling was used for authentication.
Mycoplasma contamination	All the used cell lines were negative for mycoplasma contamination.
Commonly misidentified lines<br>(See ICLAC register)	No misidentified cell lines were used.

Animals and other organisms

Policy information about studies involving animals; ARRIVE guidelines recommended for reporting animal research

Laboratory animals	7-week-old female BALB/cSLC-nu/nu and 1-month-old female CAG-rtTA3; ROSAhDX2/+ transgenic mice were used in this study. Mice were housed under ambient temperature of 24 ± 2 °C, circulating air, constant humidity of 50 ± 10% and a 12 h:12 h light/dark cycle.
Wild animals	No wild animals.
Field-collected samples	No field-collected samples.
Ethics oversight	Animal experiments were in compliance with the University Animal Care and Use Committee guidelines at Seoul National University.

Note that full information on the approval of the study protocol must also be provided in the manuscript.

Human research participants

Policy information about studies involving human research participants

Population characteristics	Ninety-nine colorectal cancer and matched normal tissue samples for patients whom underdone colorectal cancer surgery at Severance Hospital were analyzed. All patients were pathologically confirmed adenocarcinoma of the colon and rectum without preoperative treatment. Among 99 patients, 51 (51.5%) were male, and the mean age was 58.9.
Recruitment	Ninety-nine non-metastatic colorectal cancer patients without preoperative treatment who received elective colorectal cancer surgery between 2015 and 2016 at Severance Hospital were enrolled. There was no selection bias in the inclusion and exclusion criteria.
Ethics oversight	Severance Hostpital Yonsei University Health System Institutional Review Board (4-2016-0406)

Note that full information on the approval of the study protocol must also be provided in the manuscript.
